# Supplementary material for: 1H R1ρ relaxation identifies a hidden intermediate in DNA base-pairing
Source: Nat Commun. 2026 May 6;17:4114. doi: 10.1038/s41467-026-72559-6 (PMC13150003; doi:10.1038/s41467-026-72559-6)
Supplement: Supplementary file 1 — Supplementary Information [file 41467_2026_72559_MOESM1_ESM.pdf]

^{1}H $R_{1\rho}$ Relaxation Identifies a Hidden Intermediate in DNA Base-Pairing

Rubin Dasgupta[§,1,2], Christian Steinmetzger[§,1,2], Julian Ilgen[1,†], Katja Petzold[*1,2]

[1] Department of Medical Biochemistry and Biophysics, Karolinska Institutet, Solnavägen 1, 171 65 Stockholm, Sweden

[2] Department of Medical Biochemistry and Microbiology, Centre of Excellence for the Chemical Mechanisms of Life & Science for Life Laboratory, Uppsala University, Husargatan 3, 751 23, Uppsala

[§] Equal contribution

[†] Current address: Institute of Organic Chemistry, University of Regensburg, Universitätsstraße 31, 93053 Regensburg, Germany

* Correspondence to Prof. Katja Petzold, katja.petzold@imbim.uu.se

Supplementary Figures

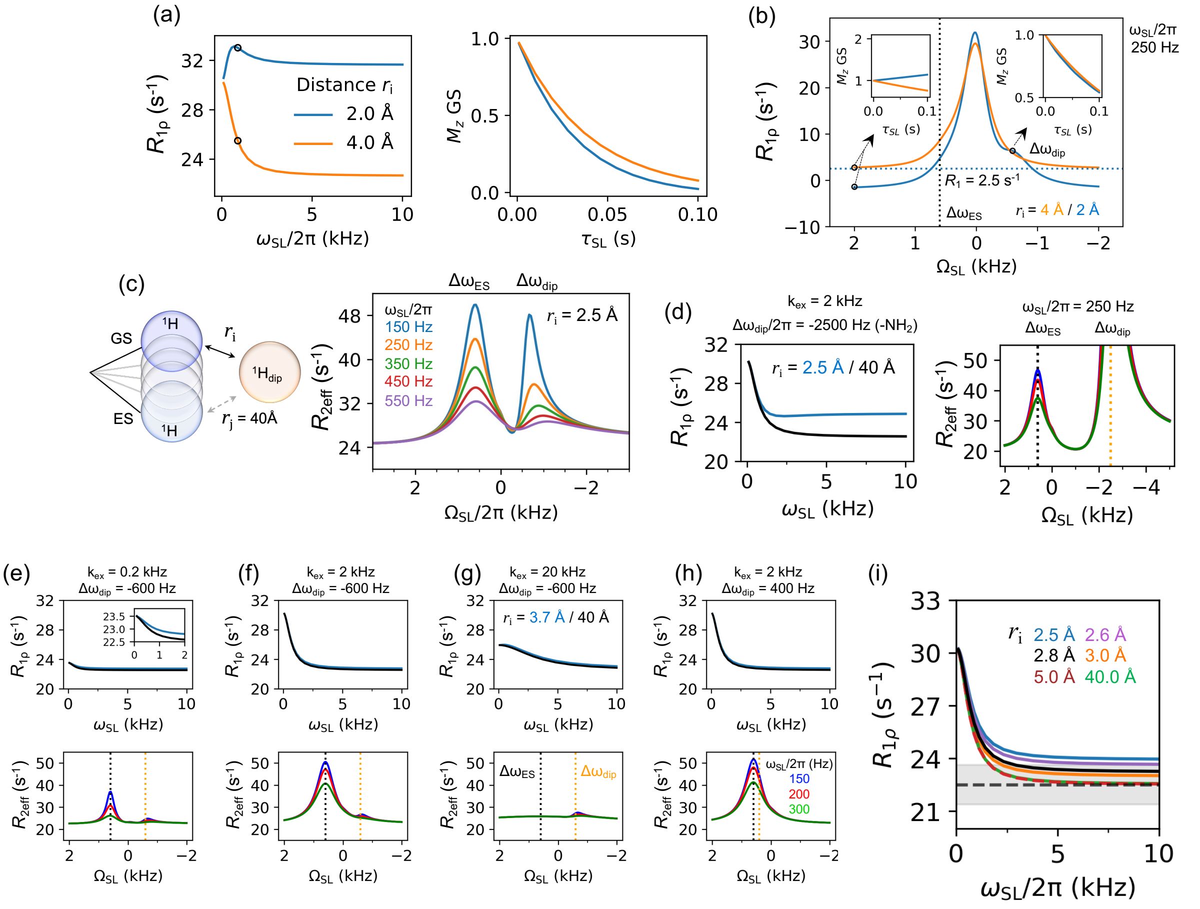

Supplementary Figure 1. (a) Representative on-resonance $R_{1\rho}$ curves (depicting an initial rise for a $^1H_{dip}$ at r_i = 2 Å (blue) as compared to r_i = 4 Å (orange), demonstrating the extent of μ contribution for smaller distances. The associated behaviour of M_Z magnetization in the GS is plotted against τ_{SL} at ω_{SL} = 200 Hz (black circles, left). (b) Simulated off-resonance $R_{1\rho}$ curves for r_j = 40 Å, with r_i = 4 Å (orange) r_i = 2 Å (blue) at ω_{SL} = 250 Hz. Inserts depict $M_{Z,\,GS}$ behaviour at offsets indicated by black circles and arrows, highlighting the rise in magnetisation at r_i = 2 Å. $\Delta\omega_{ES}$ is marked with a dotted black line while a horizontal blue dashed line indicates R_1. $\Delta\omega_{dip}$ is also denoted on the blue curve. (c) Simulated off-resonance R_{2eff} ($R_2 + R_{ex}$) at different $\omega_{SL}/2\pi$ (150 Hz blue, 250 Hz orange, 350 Hz green, 450 Hz brown and 550 Hz purple), for r_i = 2.5 Å and r_j = 40 Å (no cross-relaxation in ES). The local maximum at $\Delta\omega_{dip}$ shifts with changing ω_{SL} while $\Delta\omega_{ES}$ remains constant. Simulation parameters: k_{ex} = 2 kHz, p_{ES} = 0.5%, τ_c = 5.1 ns, R_1 = 2.5 s^{-1}, R_2 = 22.5 s^{-1}, $\Delta\omega_{ES}/2\pi$ = 600 Hz, $\Delta\omega_{dip}/2\pi$ = −600 Hz. (d) Simulation of on-resonance $R_{1\rho}$ and off-resonance R_{2eff} curves where $^1H_{dip}$ represents amino protons (-NH_2) at r_i = 2.5 Å from imino protons, as canonically observed in WCF-base-paired B-form DNA or A-form RNA helices. The average $\Delta\omega_{dip}$ between the imino and amino protons is set to be −2500 Hz with $\Delta\omega_{ES}$ = +600 Hz. The inclusion of an amino proton increases R_2 to R_2 + μ (see main text), but the large $\Delta\omega_{dip}$, minimizes any significant effect on the exchange parameters between GS and ES. (e–h) Simulated on- and off-resonance profiles for r_j = 40 Å and either r_i = 3.7 Å (light blue, representing conventional inter-imino proton distances in B-form DNA or A-form RNA) or r_i = 40 Å (black, representing no dipolar-coupled protons). Various combinations of k_{ex} and $\Delta\omega_{dip}$ were simulated. $\Delta\omega_{ES}$ and $\Delta\omega_{dip}$ are indicated with dashed black and orange lines, respectively, in the off-resonance plots. For panel (h) off-resonance curves were simulated at ω_{SL} = 150 Hz (blue), 200 Hz (red) and 300 Hz (green). The effects of $^1H_{dip}$

are negligible under most exchange conditions except when $\Delta\omega_{ES}$ and $\Delta\omega_{dip}$ have the same sign, as shown in panel (h) (see main text for details). (i) On-resonance profile using the anisotropic spectral density function from equation S30 for r_i = 2.5, 2.6, 2.8 3.0, 5.0 and 40.0 Å (blue, purple, black, orange, brown, and green) depicting that at for $r_i \geq 2.8$ Å, cross-relaxation effects remain within ± 5% (grey region, representing typical experimental error) of the R_2 rate, ensuring accurate extraction of exchange parameters.

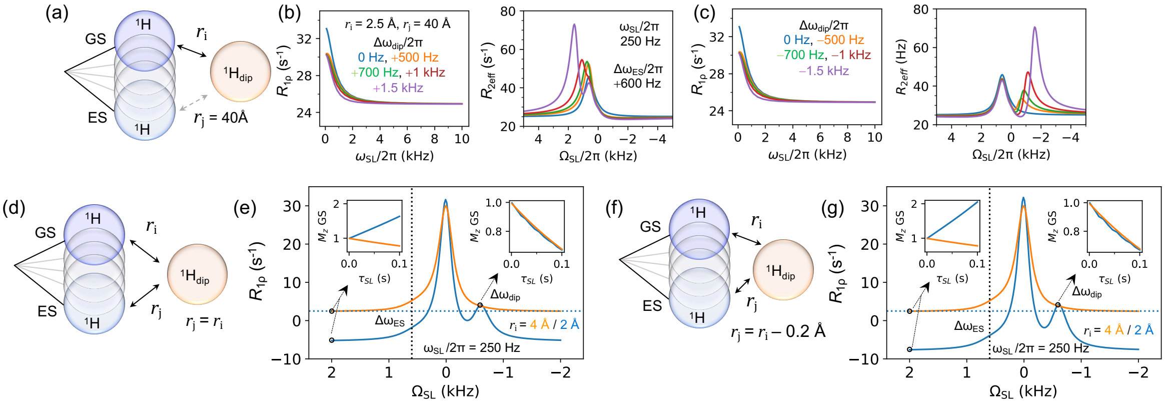

Supplementary Figure 2. (a) Model representing scenario 1 where r_i = 2.5 Å and r_j = 40 Å (no cross relaxation with ES). (b, c) Simulated on- and off-resonance profiles for various $\Delta\omega_{dip}$ values (0 Hz blue, ±500 Hz orange, ±700 Hz green, ±1 kHz brown, and ±1.5 kHz purple), with $\Delta\omega_{ES}/2\pi$ = 600 Hz and $\omega_{SL}/2\pi$ = 250 Hz. These simulations illustrate the effect of $^1H_{dip}$ at different chemical shifts of $^1H_{dip}$ relative to the excited state (ES). When $\Delta\omega_{dip}$ and $\Delta\omega_{ES}$ have opposite signs (right) exchange parameters can be estimated with high confidence; however, when their signs are same (left), accurate estimation becomes more challenging. Model representing scenario 2 (d) where r_i = 2.5 Å = r_j and scenario 3 (f) where r_i = 2.5 Å and r_j = r_i – 0.2 Å. (e, g) Simulated off-resonance $R_{1\rho}$ plots for varying distances with r_i of 4 Å (orange) or 2 Å (blue). Inserts depict the behaviour of $M_{Z,GS}$ at the offsets marked by black circles. The position of $\Delta\omega_{ES}$ is indicated with a dotted black line, while the blue dashed horizontal line marks R_1. All simulations were conducted with $\omega_{SL}/2\pi$ = 250 Hz using the parameters described in Figure S1. These plots reveal that strong dipolar coupling between $^1H_{dip}$ and both the GS and ES leads to an exponential rise in $R_{1\rho}$ rather than the expected exponential decay with respect to the spinlock duration (τ_{SL}). At extreme offsets and short distances (e.g., 2 Å), this coupling can result in apparent negative $R_{1\rho}$ values, causing the rates to deviate significantly from R_1.

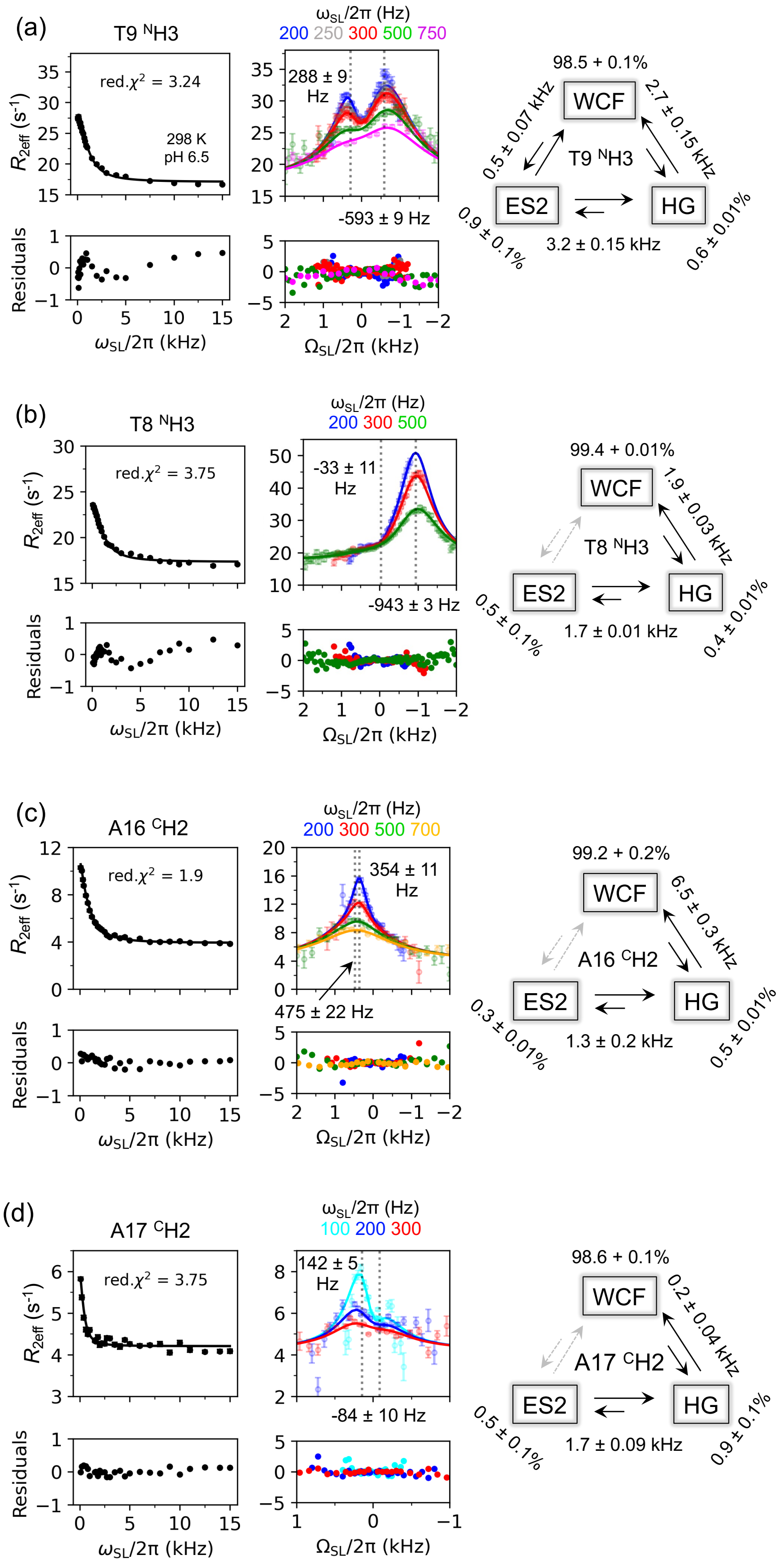
(a)
T9 NH3
red.χ2 = 3.24
298 K
pH 6.5
ωSL/2π (Hz)
200 250 300 500 750
288 ± 9 Hz
-593 ± 9 Hz
R2eff (s-1)
Residuals
ωSL/2π (kHz)
ΩSL/2π (kHz)
98.5 + 0.1%
WCF
ES2
HG
T9 NH3
0.5 ± 0.07 kHz
2.7 ± 0.15 kHz
3.2 ± 0.15 kHz
0.9 ± 0.1%
0.6 ± 0.01%
(b)
T8 NH3
red.χ2 = 3.75
ωSL/2π (Hz)
200 300 500
-33 ± 11 Hz
-943 ± 3 Hz
99.4 + 0.01%
WCF
ES2
HG
T8 NH3
1.9 ± 0.03 kHz
1.7 ± 0.01 kHz
0.5 ± 0.1%
0.4 ± 0.01%
(c)
A16 CH2
red.χ2 = 1.9
ωSL/2π (Hz)
200 300 500 700
354 ± 11 Hz
475 ± 22 Hz
99.2 + 0.2%
WCF
ES2
HG
A16 CH2
6.5 ± 0.3 kHz
1.3 ± 0.2 kHz
0.3 ± 0.01%
0.5 ± 0.01%
(d)
A17 CH2
red.χ2 = 3.75
ωSL/2π (Hz)
100 200 300
142 ± 5 Hz
-84 ± 10 Hz
98.6 + 0.1%
WCF
ES2
HG
A17 CH2
0.2 ± 0.04 kHz
1.7 ± 0.09 kHz
0.5 ± 0.1%
0.9 ± 0.1%

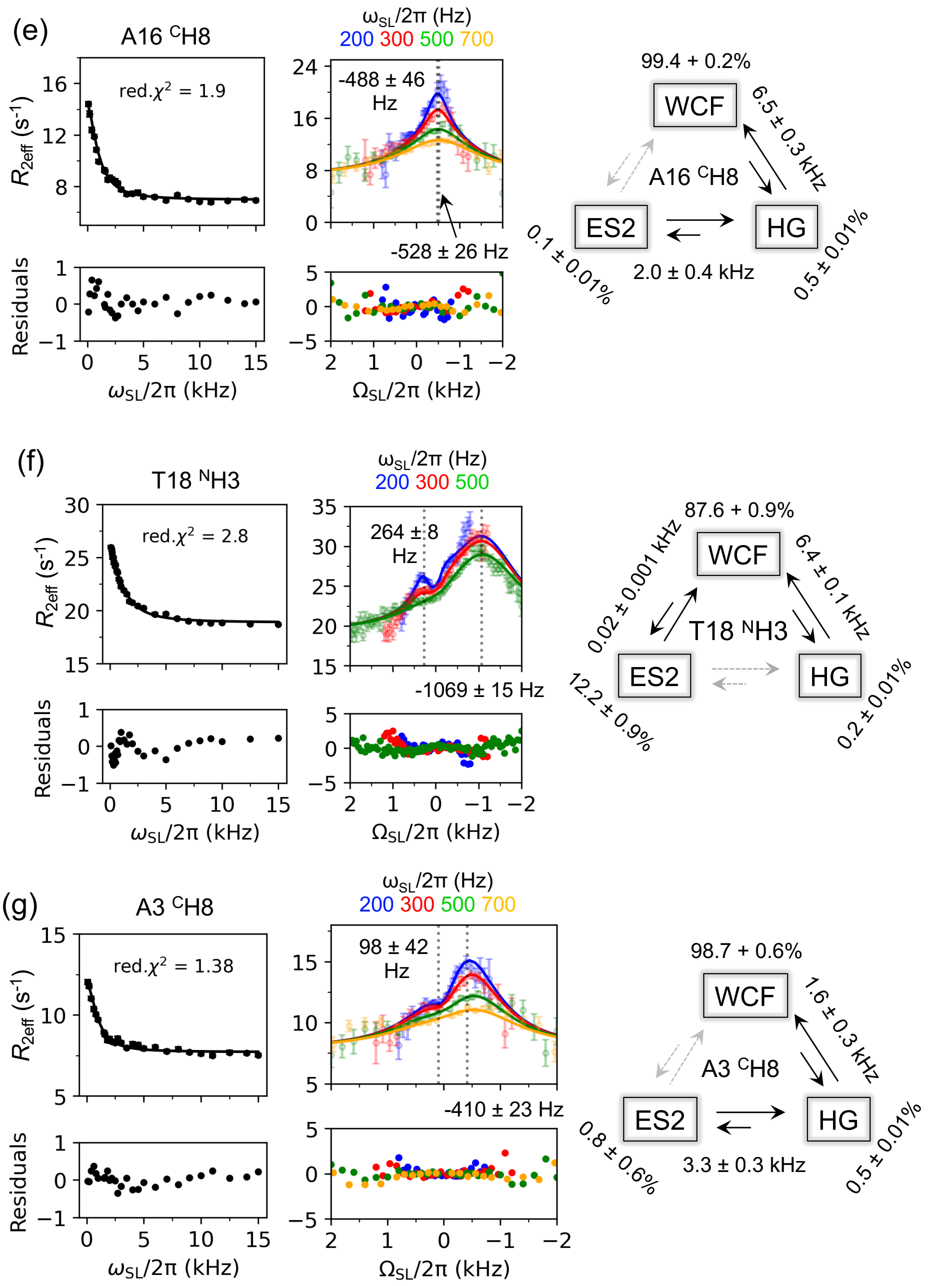

Supplementary Figure 3. ^{1}H $R_{1\rho}$ RD reveals ES2 in WCF – HG dynamics in A$_2$ DNA. On-resonance (left) and off-resonance (right) $R_{2\mathrm{eff}}$ plots for various protons (a) T9 ^{N}H3, (b) T8 ^{N}H3, (c) A16 ^{C}H2, (d) A17 ^{C}H2, (e) A16 ^{C}H8, and (f) T18 ^{N}H3, at 298 K and pH 6.5 showing three-state exchange fits to the data. Solid lines represent the fits associated with the fit parameters listed in Supplementary Table 1. $\Delta\omega_{HG}$ and $\Delta\omega_{ES2}$ are shown in the off-resonance plots, while exchange rates and populations for each conformer are shown in the schematic representation of the three-state exchange model. Global fits are shown for 1) A16 ^{C}H2 and A16 ^{C}H8 with shared k_{ex} (WCF – HG) and p_{HG} as well as for 2) T8 ^{N}H3 and A17 ^{C}H2 with shared k_{ex} (HG – ES2) and p_{ES2}. $\Delta\omega_{SL}/2\pi$ used for each off-resonance experiment are color-coded and shown above each plot. Reduced χ^2 values are indicated in the on-resonance plots with the residuals plotted below. The residuals shown below each plots depicts the quality of the fit. The error bars are estimated from 500 replicas of Monte Carlo resampling, representing mean ± 1 S.D.

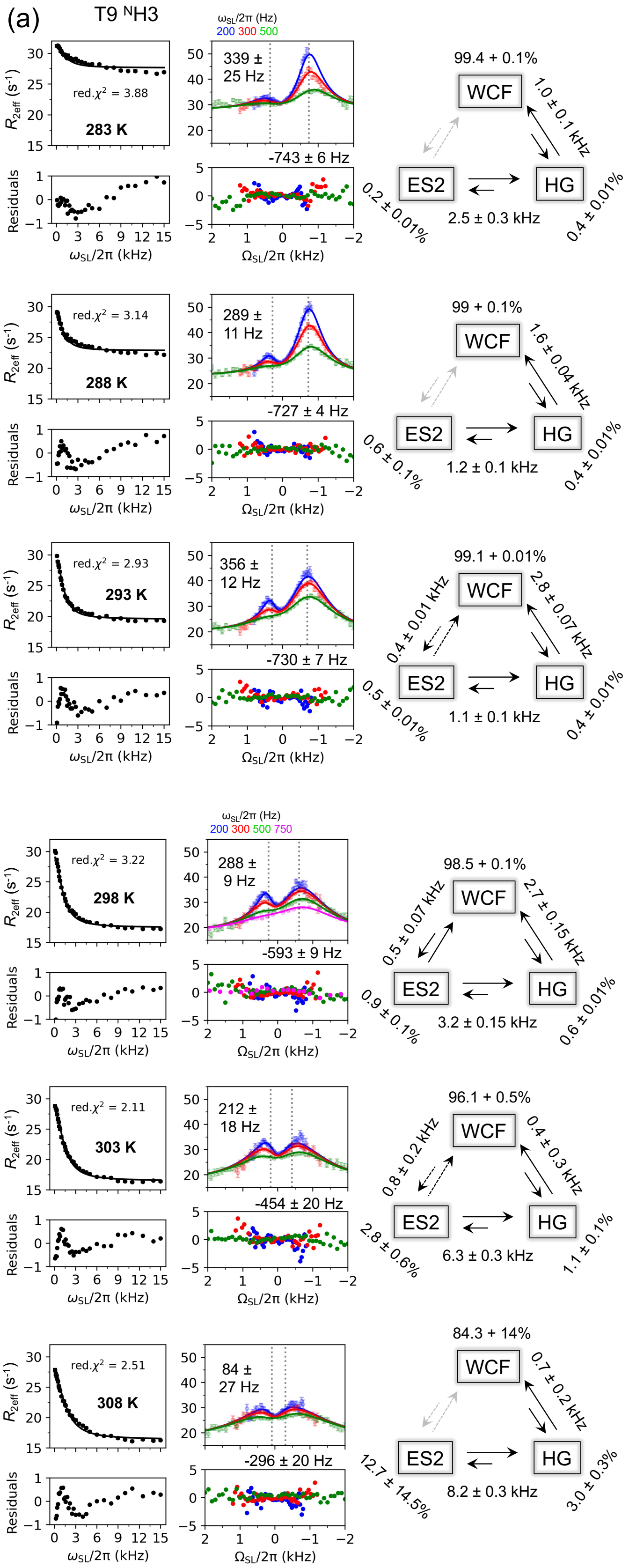

(a)
T9 NH3
ωSL/2π (Hz)
200 300 500
200 300 500 750
283 K
red.χ2 = 3.88
339 ± 25 Hz
-743 ± 6 Hz
99.4 + 0.1%
1.0 ± 0.1 kHz
0.2 ± 0.01%
2.5 ± 0.3 kHz
0.4 ± 0.01%
288 K
red.χ2 = 3.14
289 ± 11 Hz
-727 ± 4 Hz
99 + 0.1%
1.6 ± 0.04 kHz
0.6 ± 0.1%
1.2 ± 0.1 kHz
0.4 ± 0.01%
293 K
red.χ2 = 2.93
356 ± 12 Hz
-730 ± 7 Hz
99.1 + 0.01%
0.4 ± 0.01 kHz
2.8 ± 0.07 kHz
0.5 ± 0.01%
1.1 ± 0.1 kHz
0.4 ± 0.01%
298 K
red.χ2 = 3.22
288 ± 9 Hz
-593 ± 9 Hz
98.5 + 0.1%
0.5 ± 0.07 kHz
2.7 ± 0.15 kHz
0.9 ± 0.1%
3.2 ± 0.15 kHz
0.6 ± 0.01%
303 K
red.χ2 = 2.11
212 ± 18 Hz
-454 ± 20 Hz
96.1 + 0.5%
0.8 ± 0.2 kHz
0.4 ± 0.3 kHz
2.8 ± 0.6%
6.3 ± 0.3 kHz
1.1 ± 0.1%
308 K
red.χ2 = 2.51
84 ± 27 Hz
-296 ± 20 Hz
84.3 + 14%
0.7 ± 0.2 kHz
12.7 ± 14.5%
8.2 ± 0.3 kHz
3.0 ± 0.3%
WCF
ES2
HG
R2eff (s-1)
Residuals
ωSL/2π (kHz)
ΩSL/2π (kHz)

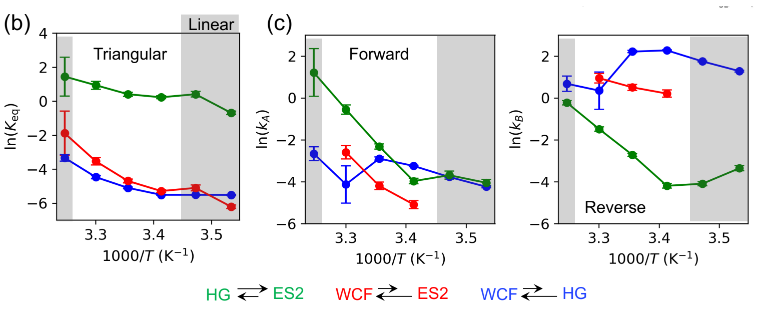

Supplementary Figure 4. (a) Temperature-dependent ^{1}H $R_{1\rho}$ RD data for T9 $^{N}H3$ in A_2 DNA obtained at temperatures ranging from 283 to 308 K in 5 K increments. The solid lines indicate fits using a three-state exchange model, with the exchange parameters and topology depicted (refer to supporting Excel file for model selection information and other fits). The $\omega_{SL}/2\pi$ used for off-resonance experiments at each temperature are color-coded. Reduced χ^2 values and corresponding temperatures are annotated on the on-resonance plots, with the fit residuals displayed below each curve. (b) The van't Hoff plots of ln(K_{eq}) vs inverse temperature with K_{eq} = p_{HG}/p_{WCF} or p_{ES2}/p_{WCF} or p_{ES2}/p_{HG} for WCF – HG (blue), WCF – ES2 (red) and HG – ES2 (green) transition. (c) Arrhenius plots for each transition, where the forward and reverse rate constants are denoted as k_A and k_B and are calculated from the exchange rates and populations. For the WCF – ES2 transition, forward and reverse rates could only be calculated for temperatures 293, 298 and 303 K because for other temperatures the experiment was not sensitive to detect this transition. This resulted in the linear topology of the three-state exchange model to be favoured for temperatures 283, 288 and 308 K by the model selection criteria (indicated in grey), largely to a too fast exchange process in relation to size of p_{ES2}. This multi-topology behaviour of the ^{1}H $R_{1\rho}$ RD temperature dependence complicates the estimation of thermodynamics parameters from either the van't Hoff or the Arrhenius plots which cannot be consolidated with a single thermodynamic model. The error bars are estimated from 500 replicas of Monte Carlo resampling, representing mean ± 1 S.D.

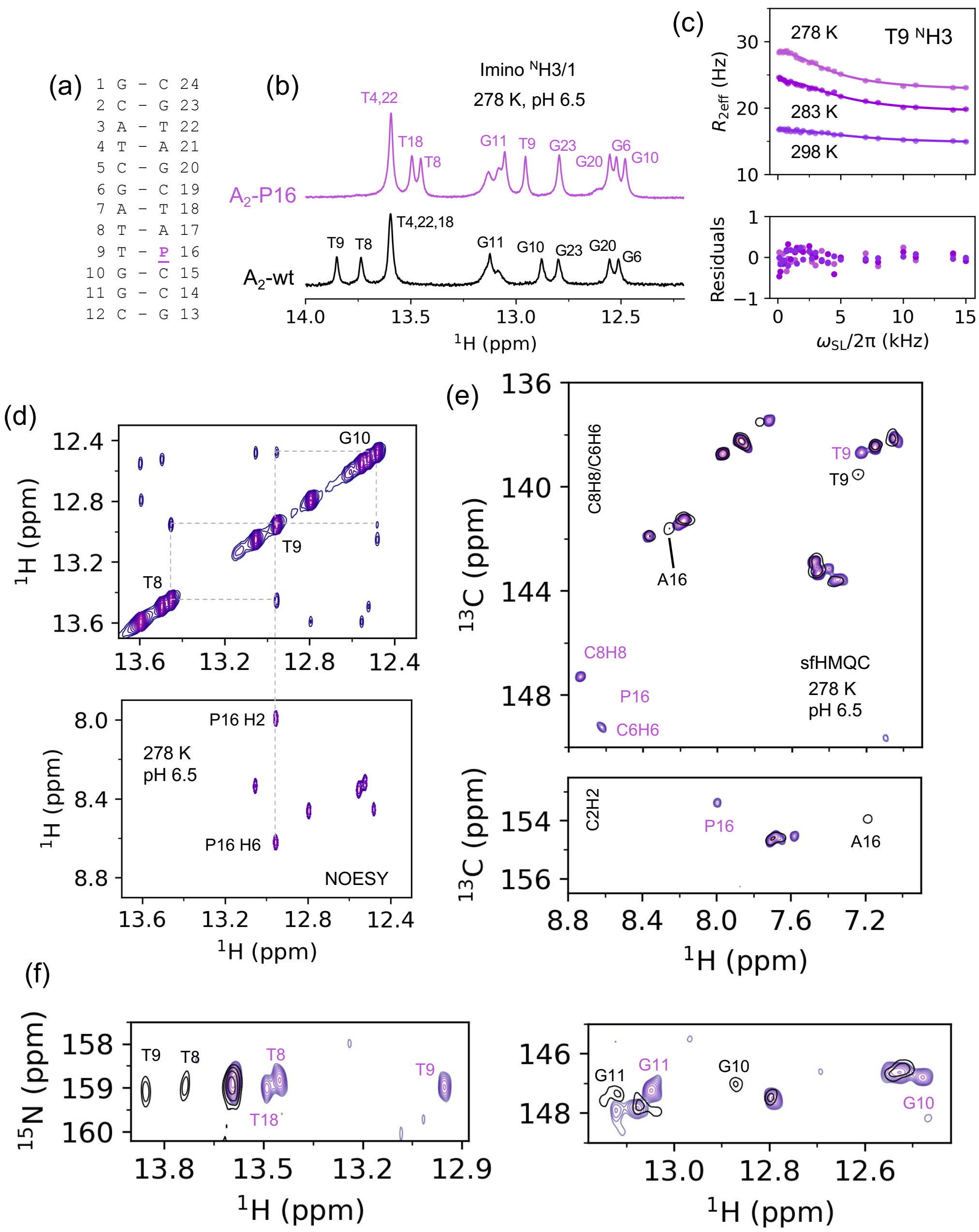

Supplementary Figure 5. **NMR of A2-P16 at 278 K and pH 6.5**. (a) Secondary structure of A2-P16 DNA where the modified base is denoted with bold violet P. (b) 1D ^{1}H imino spectrum comparing A2-P16 (violet) and A2 wt (black) with resonance assignment denoted. (c) Temperature-dependent ^{1}H $R_{1\rho}$ RD on-resonance profiles of T9 ^{N}H3 in A2-P16 are shown at 278 K, 283 K and 298 K. Solid lines show the fit of two-state reduced exchange model with the residuals plotted below with fit parameters reported in Table S4. It is evident that at 298 K the exchange rate is too fast while at 278 K the exchange regime becomes accessible to study with ^{1}H $R_{1\rho}$ RD. (d) ^{1}H–^{1}H NOESY spectrum of the imino (top) and aromatic region (bottom) with a mixing time of 180 ms. Cross peaks from T9 ^{N}H3 to P16 H2 and P16 H6 are shown to support the predominance of the WCF conformation. (e) and (f) Display the SOFAST-HMQC spectra for ^{1}H–^{13}C (C2H2, C6H6 and C8H8) and ^{1}H–^{15}N (imino), respectively, for both A2-wt (black) and A2-P16 (violet) along with the relevant assignments (Supplementary Table 7).

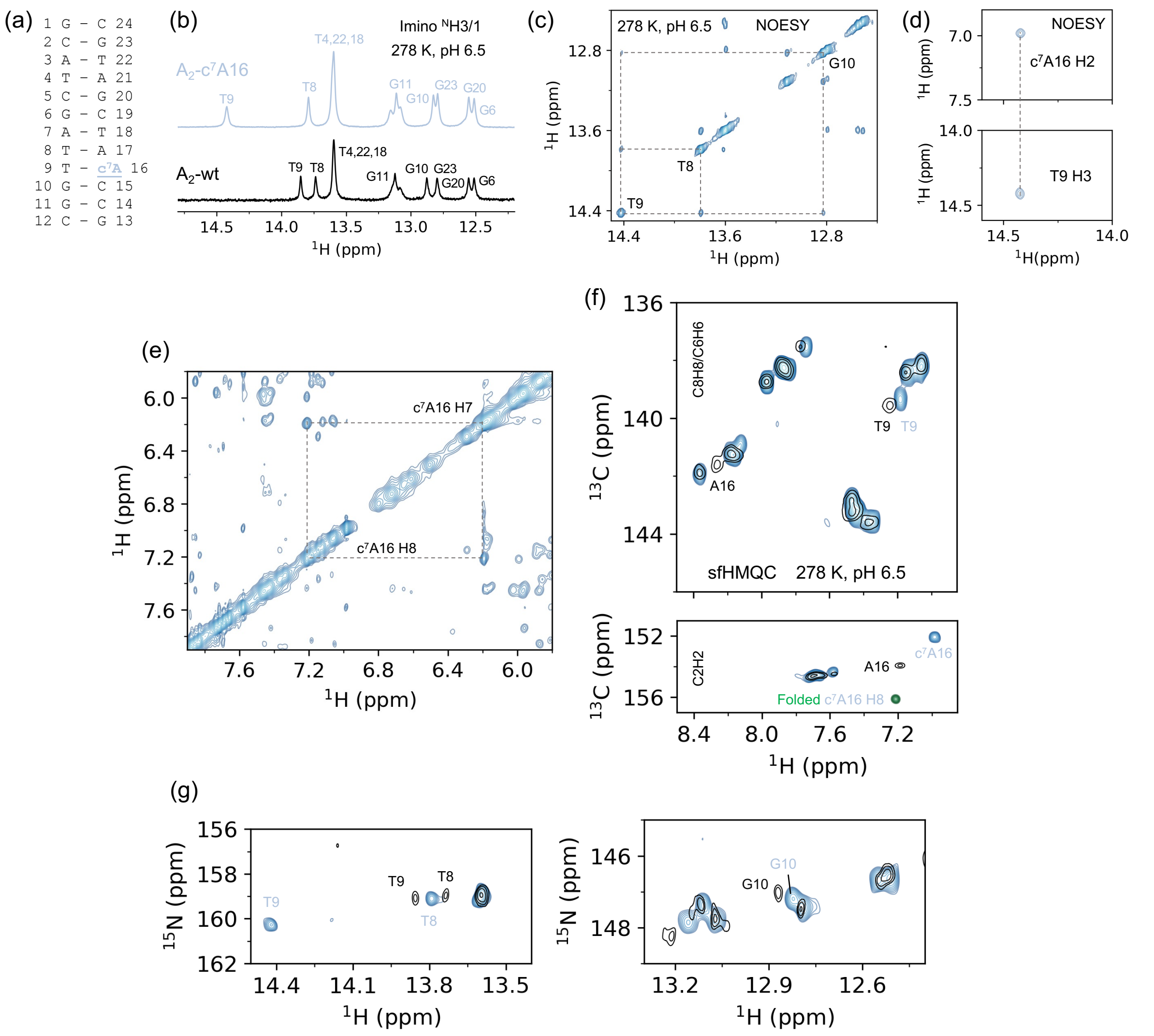

Supplementary Figure 6. **NMR of A2-c7A16 at 278 K and pH 6.5**. (a) Secondary structure of A2-c7A16 where the modified nucleotide is denoted in bold blue. (b) 1D ^{1}H imino spectrum with resonance assignment comparing A2-c7A16 (blue) and A2 wt (black). (c) ^{1}H-^{1}H NOESY spectra showing the imino walk for T8, T9 and G10 in grey dashed lines confirm the resonance assignment. (d) A NOESY cross-peak between T9 ^{N}H3 and c^{7}A16 H2 confirms WCF conformation for this base pair. (e) NOESY spectrum shows the expected cross-peaks between c^{7}A16 H8 and H7. (f) and (g) Display the SOFAST-HMQC spectra for ^{1}H–^{13}C (C2H2, C6H6 and C8H8) and ^{1}H–^{15}N (imino), respectively, for both A2-wt (black) and A2-c^{7}A16 (t blue) with partial assignment for relevant nucleotides consistent with the literature[16]. Resonance assignments are reported in Supplementary Table 8.

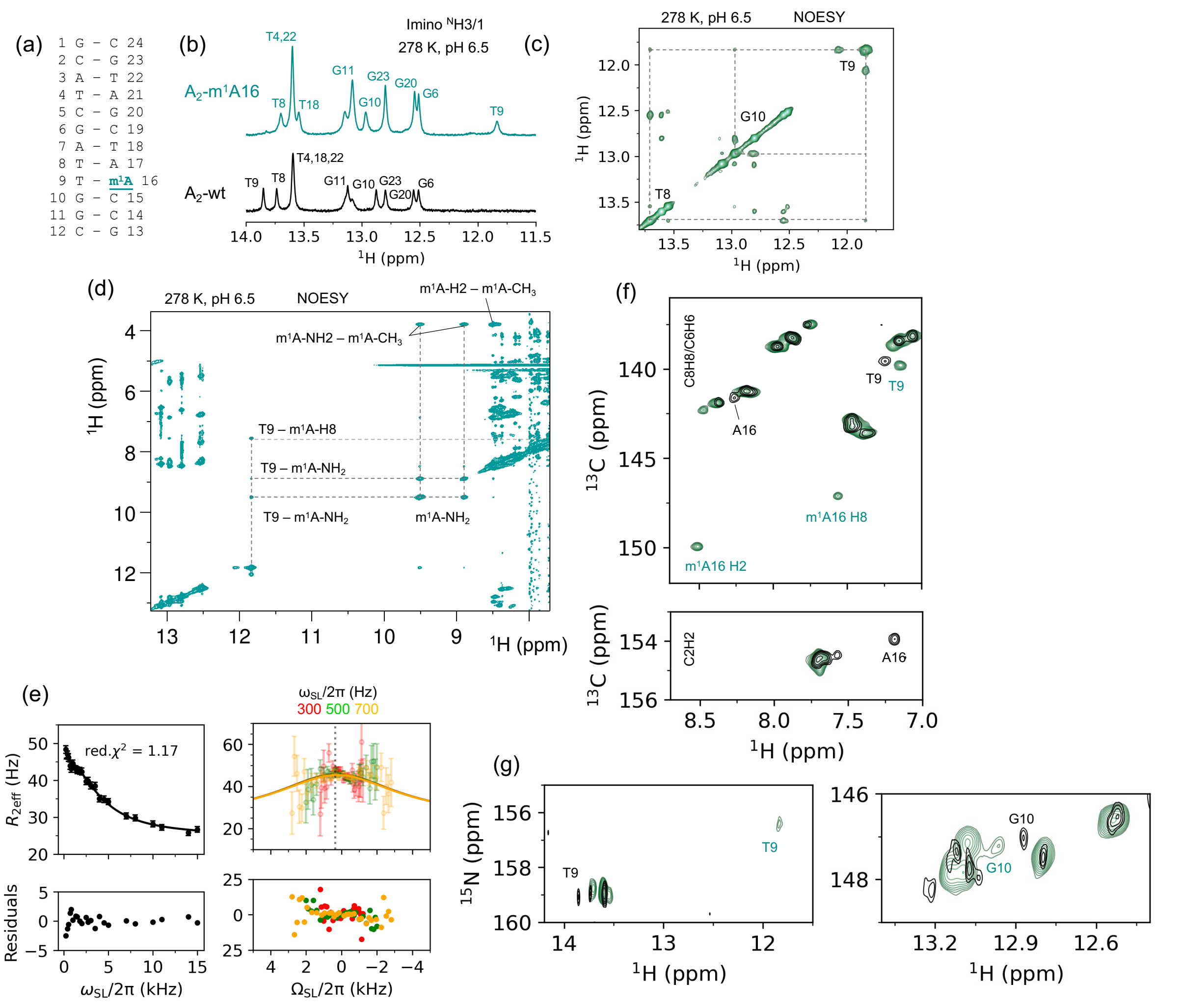

Supplementary Figure 7. NMR of A2-m¹A16 at 278 K and pH 6.5. (a) Secondary structure of A2 m¹A16 where the modified nucleotide is shown in bold green. (b) 1D ^{1}H imino spectra and resonance assignment comparing A2-m¹A16 (green) and A2-wt (black) (c) ^{1}H–^{1}H NOESY walk connecting imino protons from T8, T9 and G10 confirms the resonance assignment. (d) NOESY cross-peak between T9 ^{N}H3 and m¹A16 H8, confirming the HG conformation for this base pair. The expected cross-peaks between the m¹A –NH_2 and –CH_3 groups are also observed. (e) The ^{1}H $R_{1\rho}$ RD experiment, performed on T9 ^{N}H3 at 278 K for A2-m¹A16, shows that while the on-resonance profile displays significant relaxation dispersion, the off-resonance profile fails to capture relevant exchange parameters due to fast dynamics (Supplementary Table 4 and supporting Excel file). The solid line represents a two-state exchange model fit, with the reduced χ^2 value displayed on the on-resonance profile (right), accompanied by the residuals plotted below each curve. The dashed line on the off-resonance plot (left) represents the $\Delta\omega_{ES}$ from the two-state exchange model. The error bars are estimated from 500 replicas of Monte Carlo resampling, representing mean ± 1 S.D. (f, g) Displays the SOFAST-HMQC spectra for ^{1}H–^{15}N (imino) and ^{1}H–^{13}C (C2H2, C6H6 and C8H8) respectively, for both A2-wt (black) and A2-m¹A (green). Relevant resonance assignments (refer to Supplementary Table 9) are denoted, consistent with previous studies[9,17].

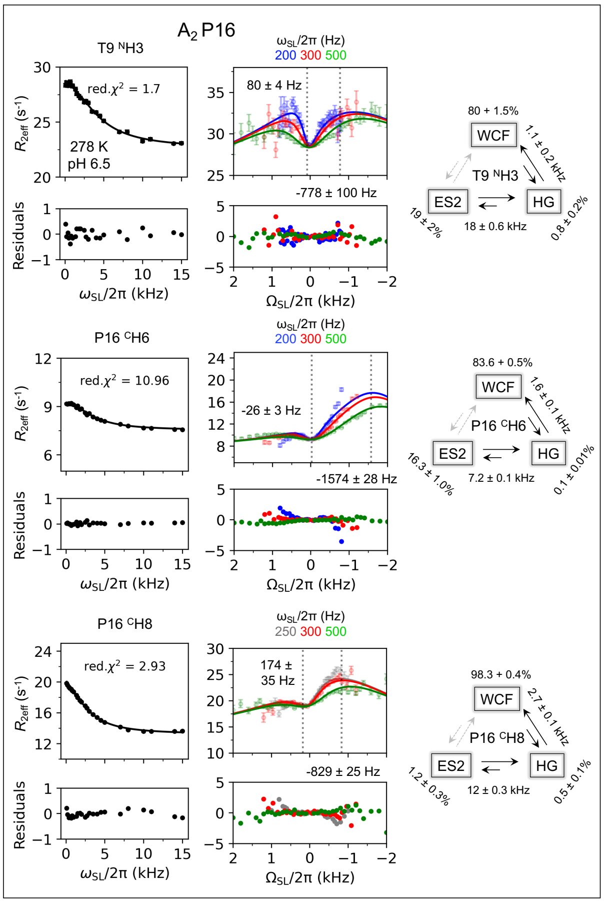

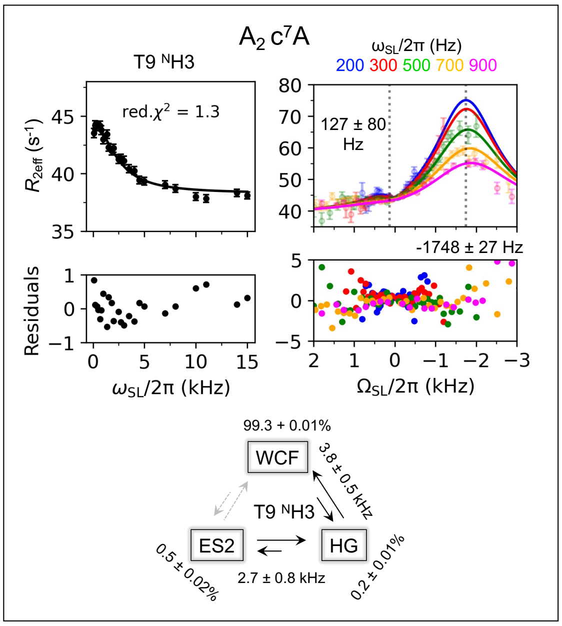

Supplementary Figure 8. Effect of modified adenine in the A16-T9 base pair on the WCF–HG–ES2 dynamics. ^{1}H $R_{1\rho}$ RD plots at 278 K and pH 6.5 for T9 ^{N}H3, P16 ^{C}H6, P16 ^{C}H8 in A_2 P16, and T9 ^{N}H3 in A_2-c^7A16. The solid lines indicate fits using a three-state linear exchange model (see supporting Excel file for model selection information). The reduced χ^2 values are shown on the on-resonance plots, while the fit residuals displayed below each curve. The values of $\Delta\omega_{HG}$ and $\Delta\omega_{ES2}$ are highlighted on the off-resonance plots with dotted

black lines. Due to high k_{ex} (HG – ES2), p_{ES2}, k_{ex} (HG – ES2), and $\Delta\omega_{ES2}$ become correlated and have larger errors than displayed (see MC plots). The exchange rates and populations are summarized in the schematic representation of the three-state exchange model. These results indicate the presence of the ES2 across both modified DNA constructs, with the detection of the HG-like state in the WCF-trapped A_2-c^7A16 duplex. The error bars are estimated from 500 replicas of Monte Carlo resampling, representing mean ± 1 S.D.

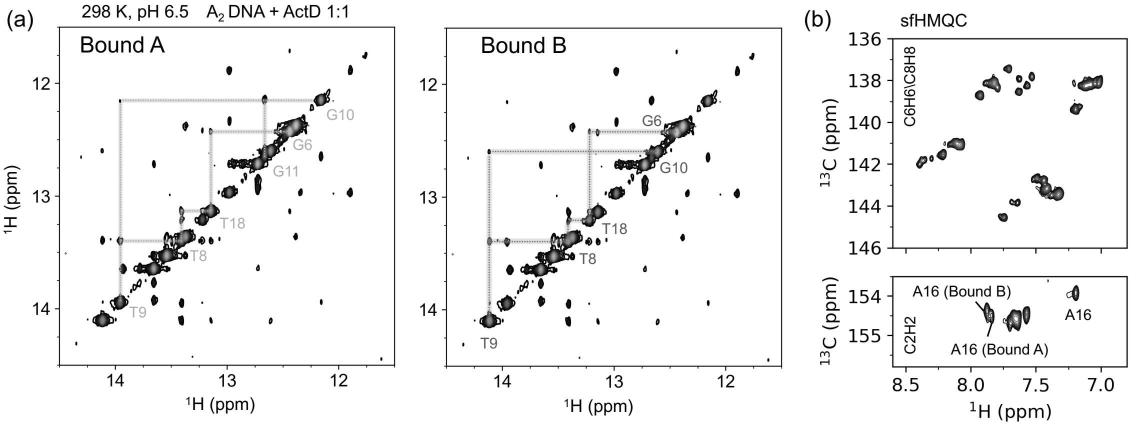

Supplementary Figure 9. **Characterization of Actinomycin D (ActD) bound A_2-DNA**. (a) Imino proton NOESY walk for the two ActD-bound fractions (Bound A and Bound B), supporting the assignment in Figure 5a. Cross-peaks confirm sequential connectivity, aiding in the identification of key resonance in the bound states. (b) ^{1}H – ^{13}C SOFAST-HMQC[18] spectrum depicting the A16 $^{C}H2$ chemical shift for both Bound A and Bound B conformations. Although spectral complexity and overlap limited full resonance assignment, the observed peaks provide partial confirmation of A16 involvement in both binding conformations.

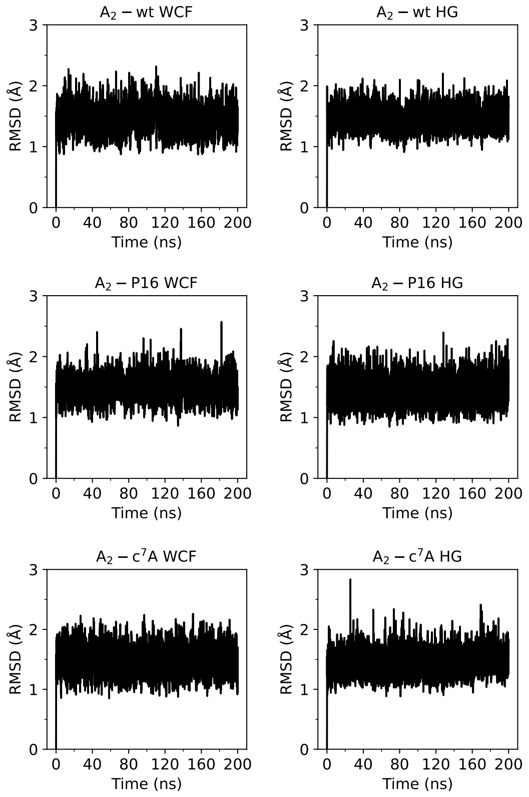

Supplementary Figure 10. **Backbone RMSD of A_2-wt, A_2-P16, and A_2-c^7A16 in WCF and HG conformations.** Root-mean-square deviations (RMSD) of the DNA backbone atoms over 200 ns molecular dynamics simulations for A_2-wt, A_2-P16, and A_2-c^7A16 in both Watson–Crick (WCF) and Hoogsteen (HG) conformations. The trajectories show overall structural stability of the DNA duplexes within the accuracy and limitations of the OL15 force field, supporting the reliability of subsequent structural and chemical shift analyses.

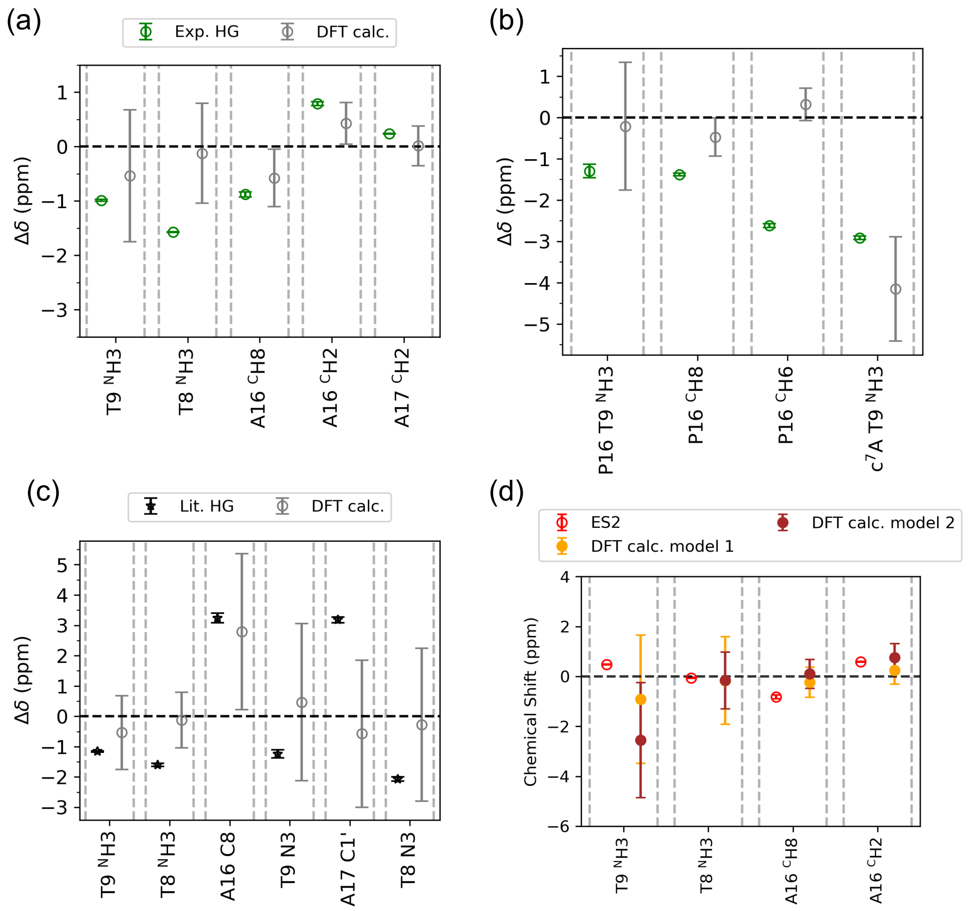

Supplementary Figure 11. Benchmarking the chemical shift calculations. (a) Relative chemical shifts (Δδ, ppm) between HG and WCF conformations obtained from experiments (green) and DFT prediction using AFNMR[19] (grey), based on 50 randomly selected frames from MD simulations. Exchangeable protons (T9 ^{N}H3, and T8 ^{N}H3) exhibit higher variability in the calculated Δδ values, whereas non-exchangeable proton (A16 ^{C}H8, A16 ^{C}H2, and A17 ^{C}H2) display more consistent predictions. (b) Δδ values for selected protons—T9 ^{N}H3, P16 ^{C}H8, and P16 ^{C}H6 in A_2-P16, and T9 ^{N}H3 in A_2-c^{7}A—show good agreement with experimental HG shifts, except for P16 ^{C}H6, for which the predicted value deviates from the observed trend. (c) Comparison of calculated Δδ values from the same 50 MD-derived frames as in (a) with literature-reported experimental chemical shifts for ^{1}H, ^{13}C, and ^{15}N nuclei[16,20,21]. (d) Δδ values between ES2 (red, empty circle) are compared with those calculated for Model 1 (orange, filled circle) and Model 2 (brown, filled circle) derived from metadynamics-based clustering with respect to WCF. Non-exchangeable protons (A16 ^{C}H8 and ^{C}H2) show relatively low variability and better alignment with experimental ES2 shifts, while exchangeable protons (T9 ^{N}H3 and T8 ^{N}H3) are less consistent. Typical error from DFT using the parameters used in the current calculation are ^{1}H (non-labile) = 0.2 to 0.3 ppm, ^{1}H (labile) ~1 ppm, ^{13}C = 2 to 3 ppm and ^{15}N = 4 to 5 ppm[19,22–24]. This shows that the errors bars observed from the ensemble structures are in line with the known deviation. Although neither model fully captures all features of ES2, additional evidence presented in the main text using modified A_2 DNA supports Model 1 as the more likely structural representation. Dots and error bars represent the difference of mean chemical shift between WCF and ES2 conformations and standard deviation via error propagation respectively obtained from the all the structures defining the ensemble of each conformation. In the DFT-predicted Δδ (ppm), dots and error bars represent the difference of mean chemical shift between WCF and HG conformations and standard deviation via error propagation respectively obtained from the all the structures defining the ensemble of each conformation.

The error bars on the calculated Δδ (ppm) in panel a, b and c represents the mean and 1x standard deviation of the randomly selected 50 frames from the MD simulations. While in panel d, the error bar is obtained from the members of clusters representing model 1 (105) and model 2 (23). The error bars in the experimental value are estimated from 500 replicas of Monte Carlo resampling, representing mean ± 1 S.D.

(a)

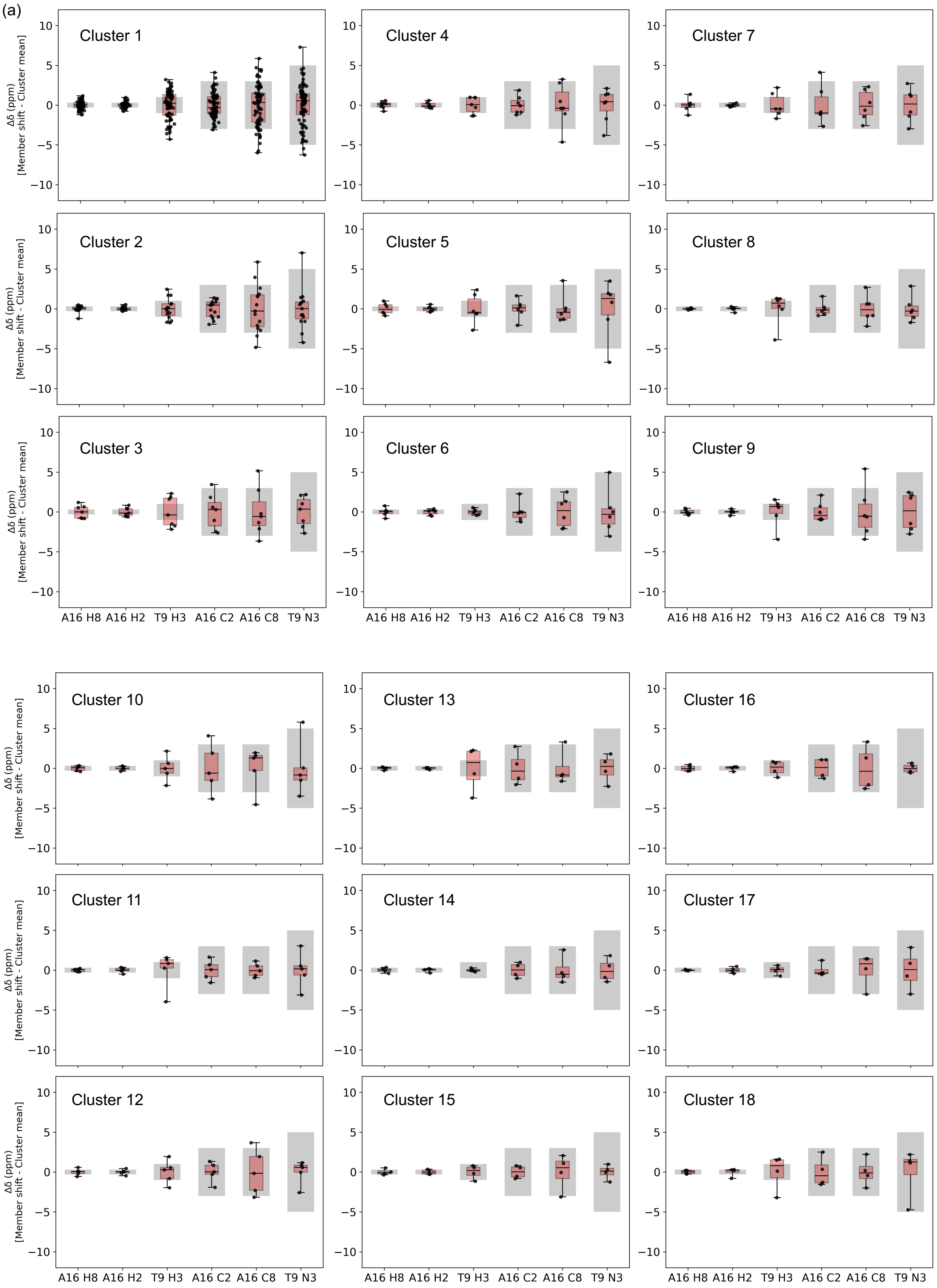
Cluster 1
Cluster 2
Cluster 3
Cluster 4
Cluster 5
Cluster 6
Cluster 7
Cluster 8
Cluster 9
Cluster 10
Cluster 11
Cluster 12
Cluster 13
Cluster 14
Cluster 15
Cluster 16
Cluster 17
Cluster 18
Δδ (ppm)
[Member shift - Cluster mean]
A16 H8
A16 H2
T9 H3
A16 C2
A16 C8
T9 N3

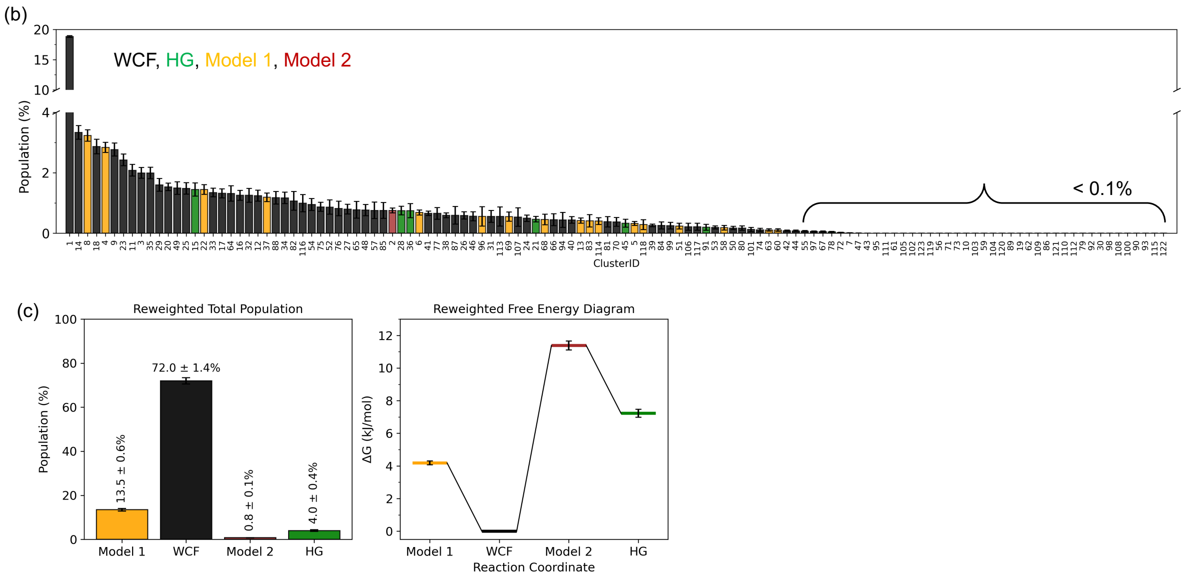

Supplementary Figure 12. **Homogeneity of 3D cluster and stability of the ensemble structures from metadynamics trajectory**. (a) AFNMR based chemical shift difference between the cluster members and the mean of the cluster of atoms typically used for $R_{1\rho}$ RD studies in an A16–T9 base-pair (T9 H3, A16 H8, A16 H2, A16 C2, A16 C8 and T9 N3) for four 3D clusters obtained from metadynamics simulation. The shaded bands reflect typical DFT errors, and the boxplots show the actual distribution of member deltas for the selected cluster and atoms. The wiskers of the boxplot are set to show the full range (min/max) of the data, and outliers are hidden for clarity. The boxes are styled with a light color and the individual member points (black dots) are overlaid with jitter for visibility. A subset of the 3D clusters (18 out of 123) is shown for clarity while the full dataset is available in Zenodo (https://doi.org/10.5281/zenodo.17155220). ~5% of the members lies beyond the typical error of DFT calculation. Given this low number of outliers the clusters are assumed to be homogenous in the chemical shift space. (b) Plot depicting reweighted, unbiased population of each 3D cluster with its statistical error, and color bars (black: WCF, green: HG, orange: Model 1 and brown: Model 2) based on agglomerative clustering of the chemical shift from the centre frame of each of the 3D cluster. The small uncertainty indicates that the metadynamics simulation provides a stable estimate of the equilibrium distribution over the sampled conformational states. The clusters representing < 0.1% of the population is denoted on the graph. Individual data points for each of the bar is deposited in Zenodo repository which can be accessed from https://doi.org/10.5281/zenodo.17155220 (c) The total population of the ensemble (right) from the combination of the 3D cluster of the metadynamics trajectory and the agglomerative clustering. The error bars are propagated from the statistical uncertainty of each cluster from panel b. Free energy diagram (left) with plateaus for each state, and error bars representing the uncertainty in free energy. The energies are shifted so that the lowest state (WCF) is at 0 kJ/mol. The error bars indicate the uncertainty in the free energy estimates, allowing for a visual comparison of the relative stabilities of the different states.

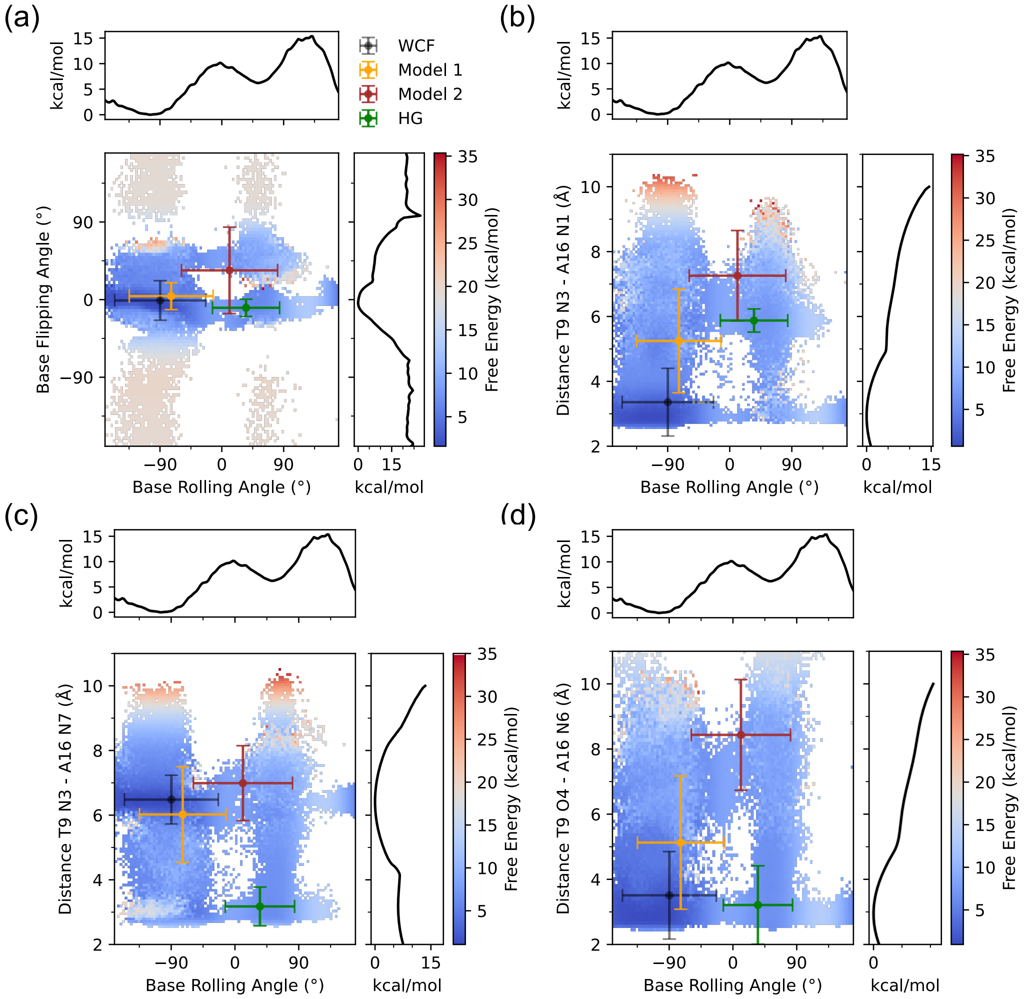

Supplementary Figure 13. 2D Free energy surfaces from metadynamics simulations. Two-dimensional free energy surfaces (2D FES) were constructed from metadynamics simulations using combinations of collective variables (CVs): base rolling angle versus (a) base flipping angle, (b) distance between T9 N3 and A16 N1, (c) distance between T9 N3 and A16 N7, and (d) distance between T9 O4 and A16 N6. Corresponding one-dimensional FES for each CV are shown along the top and right axes of each panel. Clustering based on chemical shift calculations identified four representative structural states: Watson–Crick-Franklin (WCF, black), Hoogsteen (HG, green), and two additional intermediates—Model 1 (orange) and Model 2 (brown). Error bars on the chemical shift-based cluster represents 1 standard deviation among the cluster members along the CVs. It is observed that the chemical shift clusters for WCF (black), HG (green) and Model 1 (orange) are structurally homogenous. For Model 2, the error bars are larger and show some structural inhomogeneity, however majority of the structures are localised in its unique high energy local minimum. When mapped onto the 2D FES, WCF and HG conformations occupy well-defined minima, consistent with expected stable states. Model 1 consistently localizes to a distinct low-energy basin, suggesting it represents an intermediate along the WCF–HG exchange pathway. In contrast, Model 2 resides in a higher-energy region, indicating a less favourable state compared to Model 1. These observations support Model 1 as the more likely structural candidate for the ES2 intermediate detected in experimental studies.

Supplementary Tables

Supplementary Table 1. Three-state exchange parameters for various protons from the T−A base pair in A_2 DNA. The merged cells represent parameters shared in global fitting. The definition of each parameter is given in the main text.

<table>
<tr><th></th><th>T9 ^{N}H3</th><th>A16 ^{C}H8</th><th>A16 ^{C}H2</th><th>T8 ^{N}H3</th><th>A17 ^{C}H2</th><th>T18 ^{N}H3</th><th>A3 ^{C}H8</th></tr>
<tr><td>k_{ex} (Hz)
(WC - HG)</td><td>2756 ± 152</td><td colspan="2">6568 ± 303</td><td>1915 ± 22</td><td>276 ± 43</td><td>6378 ± 128</td><td>1636 ± 276</td></tr>
<tr><td>p_{HG} (%)</td><td>0.60 ± 0.01</td><td colspan="2">0.50 ± 0.01</td><td>0.40 ± 0.01</td><td>0.90 ± 0.1</td><td>0.20 ± 0.01</td><td>0.50 ± 0.01</td></tr>
<tr><td>$\Delta\omega_{HG}$ (Hz)</td><td>−593 ± 9</td><td>−528 ± 26</td><td>475 ± 22</td><td>−943 ± 3</td><td>142 ± 5</td><td>−1069 ± 16</td><td>−410 ± 23</td></tr>
<tr><td>k_{ex} (Hz)
(WC - ES2)</td><td>501 ± 72</td><td>--</td><td>--</td><td>--</td><td>--</td><td>24 ± 0.65</td><td>--</td></tr>
<tr><td>k_{ex} (Hz)
(HG - ES2)</td><td>3277 ± 155</td><td>2034 ± 431</td><td>1360 ± 242</td><td colspan="2">1723 ± 95</td><td>--</td><td>3339 ± 265</td></tr>
<tr><td>p_{ES2} (%)</td><td>0.90 ± 0.1</td><td>0.10 ± 0.01</td><td>0.30 ± 0.01</td><td colspan="2">0.50 ± 0.10</td><td>12.20 ± 0.90</td><td>0.80 ± 0.6</td></tr>
<tr><td>$\Delta\omega_{ES2}$ (Hz)</td><td>288 ± 9</td><td>−488 ± 46</td><td>354 ± 11</td><td>−33 ± 11</td><td>−84 ± 10</td><td>264 ± 8</td><td>98 ± 42</td></tr>
<tr><td>R_1 (s^{-1})</td><td>3.50 ± 0.01</td><td>3.11 ± 0.02</td><td>1.71 ± 0.01</td><td>3.57 ± 0.01</td><td>1.70 ± 0.01</td><td>5.18 ± 0.01</td><td>3.36 ± 0.01</td></tr>
<tr><td>R_2 (s^{-1})</td><td>17.10 ± 0.06</td><td>6.96 ± 0.04</td><td>3.91 ± 0.03</td><td>17.3 ± 0.01</td><td>4.21 ± 0.01</td><td>19.00 ± 0.04</td><td>7.71 ± 0.03</td></tr>
<tr><td>Reduced χ^2</td><td>3.24</td><td colspan="2">1.90</td><td colspan="2">3.75</td><td>2.80</td><td>1.38</td></tr>
</table>

Supplementary Table 2. Sequence of unmodified and modified strand of A_2 DNA along with the respective yields from solid-phase synthesis.

DNA oligonucleotide	5'-sequence-3'	Yield (nmol)
A_2 fwd wt	GCATCGATTGGC	1073
A_2 rev P16	GCC**X**ATCGATGC	275
A_2 rev c^7A16	GCC**Y**ATCGATGC	291
A_2 rev m^1A16	GCC**Z**ATCGATGC	248
A_2 rev wt*	GCCAATCGATGC	1000

* Obtained from Integrated DNA Technologies, fwd = Forward strand, rev = Reverse strand
X = 2'-deoxynebularine (P), Y = 2'-deoxy-7-deazaadenosine (c^7A), Z = 1-methyl-2'-deoxyadenosine (m^1A)

Supplementary Table 3. Sample information for NMR spectroscopy of various DNA used in this study at pH 6.5.

DNA duplex	Conc. for NMR (mM)	Temperature
A_2-wt	1.5	288 – 308 K
A_2-P16	1.1	278 – 298 K
A_2-c^7A16	1.2	278 K
A_2-m^1A16	1.0	278 K

Supplementary Table 4. Fit parameters from temperature-dependent on-resonance ^{1}H $R_{1\rho}$ RD experiments of T9 ^{N}H3 in A$_2$ P16 fitted with a reduced two-state model[25]. Parameters for T9 ^{N}H3 in A$_2$ m^{1}A16 fitted with the two-state exchange model at 278 K is also reported.

T9 ^{N}H3	Fit parameters	298 K	283 K	278 K
A$_2$-P16	k_{ex} (Hz)	39670 ± 3255	32470 ± 1244	27084 ± 915
	Φ / $4\pi^2$ (Hz^2)	2100 ± 257	4040 ± 205	4031 ± 172
	R_2 (s^{-1})	14.6 ± 0.1	19.2 ± 0.1	22.6 ± 0.1
	Reduced χ^2	1.5	2.9	1.3
A$_2$-m^{1}A16	k_{ex} (Hz)			26205 ± 1427
	p_b (%)			13.4 ± 12.2
	$\Delta\omega$ (Hz)			347 ± 231
	R_1 (s^{-1})			5.09 ± 0.1
	R_2 (s^{-1})			25.0 ± 0.6
	Reduced χ^2			1.171

Supplementary Table 5. Fit parameters from ^{1}H $R_{1\rho}$ RD experiments for A$_2$-P16 and A$_2$-c^{7}A at 278 K and pH 6.5.

	A$_2$-P16			A$_2$-c^{7}A16
	T9 ^{N}H3	P16 ^{C}H6	P16 ^{C}H8	T9 ^{N}H3
k_{ex} (Hz) (WC - HG)	1145 ± 266	1637 ± 61	2727 ± 131	3792 ± 501
p_{HG} (%)	0.8 ± 0.2	0.1 ± 0.01	0.5 ± 0.01	0.2 ± 0.01
$\Delta\omega_{HG}$ (Hz)	−778 ± 100	−1573 ± 28	−829 ± 20	−1748 ± 27
k_{ex} (Hz) (WC - ES2)	--	--	--	--
k_{ex} (Hz) (HG - ES2)	18370 ± 612	7206 ± 133	12528 ± 9	2686 ± 893
p_{ES2} (%)	19.3 ± 2.2	16.3 ± 1	1.2 ± 0.2	0.5 ± 0.02
$\Delta\omega_{ES2}$ (Hz)	80 ± 4.5	−27 ± 3	174 ± 13	127 ± 89
R_1 (s^{-1})	3.2 ± 0.1	2.1 ± 0.01	5.0 ± 0.01	16.2 ± 0.1
R_2 (s^{-1})	22.6 ± 0.1	7.5 ± 0.01	13.2 ± 0.02	38.3 ± 0.2
Reduced χ^2	1.7	10.9	2.9	1.3

Supplementary Table 6. Spin-lock values (ω_{SL}) and frequency offsets (Ω_{SL}) with respect to the signal of interest used to acquire on- and off-resonance relaxation dispersion data. 25–35 datapoints were collected in the indicated ranges using eight spin-lock durations. An interscan delay of 1.5 s was used. The maximum spin-lock duration for each resonance was chosen such as to observe a decay to approximately 1/3 of the initial peak intensity at the lowest on-resonance ω_{SL}. A similar experimental setup was used for modified A_2 DNA at 278 K.

		ω_{SL} (Hz)	Ω_{SL} (Hz)
T8 ^{N}H3	On-res	100 to 15000	0
	Off-res	200	± 800
		300	± 1200
		500	± 2000
T9 ^{N}H3	On-res	100 to 15000	0
	Off-res	200	± 800
		250	± 1000
		300	± 1200
		500	± 2000
		750	± 3000
T18 ^{N}H3	On-res	100 to 15000	0
	Off-res	200	± 800
		300	± 1200
		500	± 2000
A16 ^{C}H2	On-res	100 to 15000	0
	Off-res	200	± 800
		300	± 1200
		500	± 2000
		700	± 2800
A16 ^{C}H8	On-res	100 to 15000	0
	Off-res	200	± 800
		300	± 1200
		500	± 2000
		700	± 2800
A17 ^{C}H2	On-res	100 to 15000	0
	Off-res	100	± 400
		200	± 800
		300	± 1200
P16 ^{C}H6	On-res	100 to 15000	0
	Off-res	200	± 800
		300	± 1200
		500	± 2000
P16 ^{C}H8	On-res	100 to 15000	0
	Off-res	200	± 800
		300	± 1200
		500	± 2000
A_2-P16 T9 ^{N}H3	On-res	100 to 15000	0
	Off-res	200	± 800
		300	± 1200
		500	± 2000
A_2-c^{7}A T9 ^{N}H3	On-res	100 to 15000	0
	Off-res	200	± 800
		300	± 1200
		500	± 2000
		700	±2800
		900	±3600

Supplementary Table 7. Chemical shift assignment in ppm for A_2-P16 at 278 K and pH 6.5

	H3	H1	H2	H8	H6	C2	C8	C6	N3	N1
G1				7.970			138.733			
C2										
A3				8.370			141.910			
T4	13.596									
C5										
G6		12.523								
A7			7.655			154.635				
T8	13.452								158.825	
T9	12.955				7.223			138.692	158.985	
G10		12.479								146.803
G11		13.053		7.721			137.476			147.250
C12										
G13										
C14										
C15										
P16			7.995	8.737	8.621	153.385	147.309	149.243		
A17			7.581			154.544				
T18	13.495								159.014	
C19										
G20		12.554								
A21										
T22	13.592									
G23		12.794								147.516
C24										

Supplementary Table 8. Chemical shift assignment in ppm for A_2-c^{7}A16 at 278 K and pH 6.5

	H3	H1	H2	H8	H6	H7	C2	C8	C6	N3	N1
G1		13.080									147.798
C2											
A3				8.366				141.927			
T4	13.598										
C5											
G6		12.513									146.628
A7											
T8	13.791									159.104	
T9	14.422				7.185				139.345	160.278	
G10		12.826									147.199
G11		13.112									147.381
C12											
G13											
C14											
C15											
c^{7}A16			6.983	7.213		6.20	152.14				
A17			7.580				154.373				
T18	13.606									159.082	
C19											
G20		12.553									146.676
A21											
T22	13.595										
G23		12.794									147.447
C24											

Supplementary Table 9. Chemical shift assignment in ppm for A_2-m[1]A16 at 278 K and pH 6.5

	H3	H1	H2	H8	H6	C2	C8	C6	N3	N1	NH_2 (1/2)	CH_3
G1		13.103										
C2												
A3				8.366			141.927					
T4	13.608											
C5												
G6		12.510								146.574		
A7												
T8	13.707								158.765			
T9	11.834				7.144			139.786	156.402			
G10		12.974								147.236		
G11		13.082								147.178		
C12												
G13		13.154								147.893		
C14												
C15												
m^1A16			8.496	7.558		149.946	147.111				9.507/8.896	3.785
A17			7.683									
T18	13.551								159.020			
C19												
G20		12.550								146.631		
A21												
T22	13.602											
G23		12.802								147.477		
C24												

Supplementary Table 10. Chemical shift assignment in ppm for A_2 DNA + ActD in a 1:1 mixture at 298 K and pH 6.5. The unbound fraction is marked in blue while the other two bound fractions are marked in red and green

	H3	H1	H2	C2
T9	13.68 / 13.98 / 14.14			
T8	13.56 / 13.43 / 13.43			
T18	13.39 / 13.17 / 13.24			
G11		13.00 / 12.69		
G10		12.75 / 12.18 / 12.63		
G6		12.41 / 12.46 / 12.46		
A16			7.24 / 7.89 / 7.92	154.06 / 154.64 / 154.51

Supplementary Table 11. Average values and the standard deviation of the collective variables used for the metadynamics simulations[26–31] for both WCF and HG conformation from unbiased 100ns MD simulation. The WCF – HG transition was probed for A16 in A_2 DNA

Collective Variables	WCF	HG
χ dihedral (radian)	−1.77 ± 0.25	0.91 ± 0.19
Base Flipping (radian)	−0.06 ± 0.08	−0.16 ± 0.05
T9 N3 – A16 N7 (Å)	6.4 ± 0.14	3.0 ± 0.31
T9 O4 – A16 N6 (Å)	3.0 ± 0.2	3.0 ± 0.30
T9 N3 – A16 N1 (Å)	3.0 ± 0.1	5.9 ± 0.2

Supplementary Note 1

Effects of cross-relaxations on the $R_{1\rho}$ rates without chemical exchange

The Bloch equation to model the magnetization behaviour under a spin-lock for a single spin in the rotating frame is given by[1]

$$\frac{\mathrm{d}}{\mathrm{d}t}\begin{bmatrix} \mathrm{M}_x(t) \\ \mathrm{M}_y(t) \\ \mathrm{M}_z(t) \end{bmatrix} = -1\begin{bmatrix} R_2 & \Omega & 0 \\ -\Omega & R_2 & \omega_{\mathrm{SL}} \\ 0 & -\omega_{\mathrm{SL}} & R_1 \end{bmatrix}\begin{bmatrix} \mathrm{M}_x(0) \\ \mathrm{M}_y(0) \\ \mathrm{M}_z(0) \end{bmatrix} \quad (1)$$

Where M_X, M_Y and M_Z represents the bulk magnetization along the X, Y and Z axis, R_2 and R_1 are the transversal and longitudinal auto-relaxation rates respectively. The off-diagonal terms describe the precession frequency Ω along the Z-axis and spin-lock field ω_{SL} along the X-axis. Rotating equation S1 to the spin-locking field or the double rotating frame around Y-axis by an angle θ with respect to Z-axis, provides the rate equation for the magnetization along each axis.

$$\frac{\mathrm{d}}{\mathrm{d}t}\begin{bmatrix} \mathrm{M}'_\mathrm{X}(t) \\ \mathrm{M}'_\mathrm{Y}(t) \\ \mathrm{M}'_\mathrm{Z}(t) \end{bmatrix} = -1\begin{bmatrix} R_1\sin^2\theta + R_2\cos^2\theta & \sqrt{\Omega^2+\omega^2} & (R_2-R_1)\sin\theta\cos\theta \\ -\sqrt{\Omega^2+\omega^2} & R_2 & 0 \\ (R_2-R_1)\sin\theta\cos\theta & 0 & R_1\cos^2\theta + R_2\sin^2\theta \end{bmatrix}\begin{bmatrix} \mathrm{M}'_\mathrm{X}(0) \\ \mathrm{M}'_\mathrm{Y}(0) \\ \mathrm{M}'_\mathrm{Z}(0) \end{bmatrix} \quad (2)$$

Under the experimentally condition of $\omega_{SL} >> (R_2 - R_1)\sin\theta\cos\theta$ and due to rapid inter-conversion of Mx' and My' because of $\sqrt{\Omega^2+\omega^2}$, the off-diagonal term $(R_2 - R_1)\sin\theta\cos\theta$ becomes 0. This provides the rate equation for the Mz' under the spin-locking field

$$\frac{\mathrm{d}}{\mathrm{d}t}\,[\mathrm{M}'_\mathrm{Z}(t)] = -R_{1\rho}\mathrm{M}'_\mathrm{Z}(0) \quad (3)$$

The general solution to equation S3 is

$$\mathrm{M}'_\mathrm{Z}(t) = \exp\,(-R_{1\rho}t)\mathrm{M}'_\mathrm{Z}(0) \quad (4)$$

here, t is the duration of the spin-locking field which can be represented as τ_{SL} and $R_{1\rho}$ is given by

$$R_{1\rho} = R_1\cos^2\theta + R_2\sin^2\theta \quad (5)$$

This equation shows that the Mz' magnetization will decay monoexponentially with the rate of $R_{1\rho}$ which is then given as the weighted sum of the R_1 and R_2 rates. To ascertain the effect of longitudinal and transverse cross-relaxation rates (σ and μ) between two spins (a and b), the matrix in equation S1 is expanded to

$$\frac{\mathrm{d}}{\mathrm{d}t}\begin{bmatrix} \mathrm{Ma_X}(t) \\ \mathrm{Ma_Y}(t) \\ \mathrm{Ma_Z}(t) \\ \mathrm{Mb_X}(t) \\ \mathrm{Mb_Y}(t) \\ \mathrm{Mb_Z}(t) \end{bmatrix} = -1\begin{bmatrix} R_{2a} & \Omega & 0 & \mu & 0 & 0 \\ -\Omega & R_{2a} & \omega_{\mathrm{SL}} & 0 & \mu & 0 \\ 0 & -\omega_{\mathrm{SL}} & R_{1a} & 0 & 0 & \sigma \\ \mu & 0 & 0 & R_{2b} & \Omega & 0 \\ 0 & \mu & 0 & -\Omega & R_{2b} & \omega_{\mathrm{SL}} \\ 0 & 0 & \sigma & 0 & -\omega_{\mathrm{SL}} & R_{2b} \end{bmatrix}\begin{bmatrix} \mathrm{Ma_X}(0) \\ \mathrm{Ma_Y}(0) \\ \mathrm{Ma_Z}(0) \\ \mathrm{Mb_X}(0) \\ \mathrm{Mb_Y}(0) \\ \mathrm{Mb_Z}(0) \end{bmatrix} \quad (6)$$

Here,

$$\mu = \mathrm{D}\,(2\mathrm{J}(0) + 3\mathrm{J}(\omega)) \quad \text{and} \quad \sigma = \mathrm{D}\,(-\mathrm{J}(0) + 6\mathrm{J}(2\omega))$$

$$\mathrm{D} = \frac{1}{4}\left(\frac{\gamma_H^2\hbar\mu_0}{4\pi}\right)^2\frac{1}{r^6} \quad \text{and} \quad \mathrm{J}(\omega) = \frac{2}{5}\frac{\tau_c}{(1+\omega^2\tau_c^2)} \quad (7)$$

Where τ_c is the rotation correlation time, ω is the proton Larmor frequency, γ_H is the proton gyromagnetic ratio, $\hbar$ is the reduced Planck's constant, μ_0 is the vacuum permeability, and r is the inter-proton distance. Rotating the matrix in equation S6 around y-axis into the double rotating frame, the $R_{1\rho}$ is given as

$$R_{1\rho} = (R_1 + \sigma)\cos^2\theta + (R_2 + \mu)\sin^2\theta \tag{8}$$

This suggests that the cross-relaxation rates will scale the R_1 and R_2 contribution to the $R_{1\rho}$ rate but do not affect the mono-exponential behaviour of the Z-magnetization decay under spin-locking field. Equation S8 can be now expanded to include chemical exchange as shown below.

Bloch-McConnel (BM) matrix for two-state exchange

The BM matrix for a two-state chemical ES (b) ⇌ GS (a) exchange is given by[1]

$$\frac{\mathrm{d}}{\mathrm{d}t}\begin{bmatrix}\mathbf{M}_\mathrm{a}(t)\\ \mathbf{M}_\mathrm{b}(t)\end{bmatrix} = \left\{\begin{bmatrix}\mathbf{L}_\mathrm{a} & \mathbf{0}\\ \mathbf{0} & \mathbf{L}_\mathrm{b}\end{bmatrix} + \mathbf{K}\otimes\mathbf{1}\right\}\begin{bmatrix}\mathbf{M}_\mathrm{a}(0)\\ \mathbf{M}_\mathrm{b}(0)\end{bmatrix} \tag{9}$$

$$\mathbf{L}_{i(a,b)} = -1\begin{bmatrix} R_{2i} & \delta_i & -\omega_\mathrm{ramp}\\ -\delta_i & R_{2i} & \omega_\mathrm{SL}\\ \omega_\mathrm{ramp} & -\omega_\mathrm{SL} & R_{1i}\end{bmatrix} \qquad \begin{aligned}\delta_\mathrm{a} &= -p_\mathrm{b}\,\Delta\omega_\mathrm{b} - \Omega_\mathrm{SL}\\ \delta_\mathrm{b} &= p_\mathrm{a}\,\Delta\omega_\mathrm{b} - \Omega_\mathrm{SL}\end{aligned} \tag{10}$$

$$\mathbf{K} = \begin{bmatrix}-k_{12} & k_{21}\\ k_{12} & -k_{21}\end{bmatrix} \qquad \begin{aligned}k_{12} &= p_\mathrm{b}k_\mathrm{ex}\\ k_{21} &= p_\mathrm{a}k_\mathrm{ex}\end{aligned} \tag{11}$$

$$[\mathbf{M}_\mathrm{a}(0) \quad \mathbf{M}_\mathrm{b}(0)]^\mathrm{T} = \begin{bmatrix}\mathrm{M_{X(a)}} & \mathrm{M_{Y(a)}} & \mathrm{M_{Z(a)}} & \mathrm{M_{X(b)}} & \mathrm{M_{Y(b)}} & \mathrm{M_{Z(b)}}\end{bmatrix}^\mathrm{T} \tag{12}$$

Where R_2 is the transverse relaxation rate, R_1 is the longitudinal relaxation rate, p_a is the population of the GS, p_b is the population of the ES, k_ex ($k_\mathrm{ab} + k_\mathrm{ba}$) is the exchange rate, $\Delta\omega_\mathrm{b}$ is the offset of the ES with respect to the observed signal, Ω_SL is the offset of the spin-lock with respect to the observed signal, ω_SL is the spin-lock strength and ω_ramp is the strength of the ramp pulse flanking the spin-lock. The superscript $^\mathrm{T}$ denotes the transpose of the initial magnetization vector. $\mathbf{0}$ and $\mathbf{1}$ are the 3 x 3 zero and identity matrix, respectively. The solution to equation S9 is given by,

$$\begin{bmatrix}\mathbf{M}_\mathrm{a}(t)\\ \mathbf{M}_\mathrm{b}(t)\end{bmatrix} = \exp\left(\left\{\begin{bmatrix}\mathbf{L}_\mathrm{a} & \mathbf{0}\\ \mathbf{0} & \mathbf{L}_\mathrm{b}\end{bmatrix} + \mathbf{K}\otimes\mathbf{1}\right\} * \tau_\mathrm{SL}\right)\cdot\begin{bmatrix}\mathbf{M}_\mathrm{a}(0)\\ \mathbf{M}_\mathrm{b}(0)\end{bmatrix} \tag{13}$$

Where τ_{SL} is the duration of the spinlock. Assuming that, $R_{1\mathrm{a}} = R_{1\mathrm{b}}$ and $R_{2\mathrm{a}} = R_{2\mathrm{b}}$, $R_{1\rho}$ is estimated by fitting a mono-exponential decay to

$$M_{\mathrm{Z}(a)}(t) = M_{\mathrm{Z}(a)}(0)\exp\left(-R_{1\rho} * \tau_\mathrm{SL}\right) \tag{14}$$

with, $M_{\mathrm{Z}(a)}(0) = p_a$. The analytical approximation of the BM matrix to describe $R_{1\rho}$ was derived to be[1,2]:

$$R_{1\rho} = R_1\cos^2\theta + R_2\sin^2\theta + R_{ex}\sin^2\theta \tag{15}$$

Here, $\theta = \arctan\left(\frac{\omega_\mathrm{SL}}{\Omega_\mathrm{SL}}\right)$. Rearranging equation S15 gives the expression for the $R_{2\mathrm{eff}}$ or $R_2 + R_\mathrm{ex}$,

$$R_{2\mathrm{eff}} = R_2 + R_\mathrm{ex} = \frac{R_{1\rho} - R_1\cos^2\theta}{\sin^2\theta} \tag{16}$$

Extended two-state Bloch-McConnell matrix

Equation S9 can be extended to include cross-relaxation, and can be written as[3]:

$$\frac{\mathrm{d}}{\mathrm{d}t}\begin{bmatrix}\mathbf{M}_\mathrm{a}(t)\\ \mathbf{M}_\mathrm{b}(t)\\ \mathbf{M}_\mathrm{c}(t)\end{bmatrix} = \left\{\begin{bmatrix}\mathbf{L}_\mathrm{a} & \mathbf{0} & \mathbf{L}_\mathrm{CR}\\ \mathbf{0} & \mathbf{L}_\mathrm{b} & \mathbf{L}_\mathrm{CR}\\ \mathbf{L}_\mathrm{CR} & \mathbf{L}_\mathrm{CR} & \mathbf{L}_\mathrm{c}\end{bmatrix} + \mathbf{K}\otimes\mathbf{1}\right\}\begin{bmatrix}\mathbf{M}_\mathrm{a}(0)\\ \mathbf{M}_\mathrm{b}(0)\\ \mathbf{M}_\mathrm{c}(0)\end{bmatrix} \tag{17}$$

Here, $\mathbf{M_c}$ is the magnetization vector for $^1\mathrm{H}_\mathrm{dip}$ with the cross-relaxation given by matrix $\mathbf{L_{CR}}$.

$$\mathbf{L}_{\mathrm{CR}} = -1\begin{bmatrix} \mu & 0 & 0 \\ 0 & \mu & 0 \\ 0 & 0 & \sigma \end{bmatrix} \tag{18}$$

The distance from GS and ES to the dipolar proton can be modulated using equation S17 and S18. The $R_{1\rho}$ is given by

$$R_{1\rho} = (R_1 + \sigma)\cos^2\theta + (R_2 + \mu)\sin^2\theta + R_{ex}\sin^2\theta \tag{19}$$

This, again, describes a mono-exponential decay of spin-locked magnetization in the presence of cross-relaxation.

Three-state exchange model without cross-relaxation

The three-state exchange with was modelled using the following Bloch-McConnell matrix[1]:

$$\frac{\mathrm{d}}{\mathrm{d}t}\begin{bmatrix}\mathbf{M}_a(t)\\ \mathbf{M}_b(t)\\ \mathbf{M}_c(t)\end{bmatrix} = \left\{\begin{bmatrix}\mathbf{L}_a & \mathbf{0} & \mathbf{0}\\ \mathbf{0} & \mathbf{L}_b & \mathbf{0}\\ \mathbf{0} & \mathbf{0} & \mathbf{L}_c\end{bmatrix} + \mathbf{K}\otimes\mathbf{1}\right\}\begin{bmatrix}\mathbf{M}_a(0)\\ \mathbf{M}_b(0)\\ \mathbf{M}_c(0)\end{bmatrix} \tag{20}$$

$$\mathbf{L}_{\mathrm{i(a,b,c)}} = -1\begin{bmatrix} R_{2i} & \Delta_i & -\omega_{ramp}\\ -\Delta_i & R_{2i} & \omega_{\mathrm{SL}}\\ \omega_{ramp} & -\omega_{\mathrm{SL}} & R_{1i}\end{bmatrix} \qquad \begin{aligned}\Delta_a &= -p_b\Delta\omega_b - p_c\Delta\omega_c - \Omega_{\mathrm{SL}}\\ \Delta_b &= (1-p_b\Delta\omega_b) - p_c\Delta\omega_c - \Omega_{\mathrm{SL}}\\ \Delta_c &= (1-p_c)\Delta\omega_c - p_b\Delta\omega_b - \Omega_{\mathrm{SL}}\end{aligned} \tag{21}$$

Different topologies of the three-state exchange can be modelled by changing the rate matrix **K**. For a general triangular topology (ES1 (b) ⇌ GS (a) ⇌ ES2 (c) ⇌ ES1 (b)), the K matrix is given by:

$$\mathbf{K} = \begin{bmatrix} -k_{12}-k_{13} & k_{21} & k_{31}\\ k_{12} & -k_{21}-k_{23} & k_{32}\\ k_{13} & k_{23} & -k_{31}-k_{32}\end{bmatrix} \tag{22}$$

$$\begin{aligned} k_{12} &= k_{ex\_ab}\frac{p_b}{p_a+p_b} & k_{21} &= k_{ex\_ab}\frac{p_a}{p_a+p_b}\\ k_{13} &= k_{ex\_ac}\frac{p_c}{p_a+p_c} & k_{31} &= k_{ex\_ac}\frac{p_a}{p_a+p_c}\\ k_{23} &= k_{ex\_bc}\frac{p_c}{p_b+p_c} & k_{32} &= k_{ex\_bc}\frac{p_b}{p_b+p_c}\end{aligned} \tag{23}$$

For a three-state linear topology (GS (a) ⇌ ES1 (b) ⇌ ES2 (c)), k_{31} and k_{13} = 0, while for the star-like topology (ES1 (b) ⇌ GS (a) ⇌ ES2 (c)), k_{32} and k_{23} = 0.

Spectral density function for axially symmetric molecule (a case of 12 base-pair A_2 DNA)

The spectral density defined in equation S7 is generally used for rigid sphere and is normally applied to the NMR relaxation study of wide range of biomolecules. However, given the cylindrical nature of the A_2 DNA used in this study, this spectral density was expanded to use definitions of an axially symmetric rod like particle. The correlation function for such a particle is given by[4]:

$$C_0(t) = A_1\exp\left(-\frac{t}{\tau_1}\right) + A_2\exp\left(-\frac{t}{\tau_2}\right) + A_3\exp\left(-\frac{t}{\tau_3}\right) \tag{24}$$

With,

$$\begin{gathered} A_1 = (1.5\cos^2\alpha - 0.5)^2, A_2 = 3\sin^2\alpha\cos^2\alpha, A_3 = 0.75\sin^4\alpha\,; \alpha = \frac{\pi}{2}\\ \tau_1 = \frac{1}{6D_\perp}, \tau_2 = \frac{1}{D_\parallel + 5D_\perp}, \tau_3 = \frac{1}{4D_\parallel + 2D_\perp}\end{gathered} \tag{25}$$

The α is the angle between the imino N-H bond vector and the DNA helical axis. For a canonical B-form helix the α is π/2 with a slight deviation of 1.2° which is negligible. $D_\parallel$ and $D_\perp$ are the longitudinal and transverse diffusion constants and are given by[5–7]

$$D_\perp = \frac{3k_BT\left(\ln(p) - 0.662 + \frac{0.917}{p} - \frac{0.050}{p^2}\right)}{\pi\eta_0 L^3} \tag{26}$$

$$D_{\parallel} = \frac{4k_BTp^2}{3.84\pi\eta_0 L^3\left(1 + \frac{0.677}{p} - \frac{0.183}{p^2}\right)} \tag{27}$$

Where, k_B is the Boltzmann constant, T is the temperature, η_0 is the viscosity of solvent, L is the length of the DNA and p is the aspect ratio given by[5–7] $p = L/2R$ with R being the radius of the DNA. The average diffusion constant is given by:

$$D = \frac{D_{\parallel} + 2D_{\perp}}{3} \tag{28}$$

The effect of fast internal motions of the time scale τ_e when $\tau_e << (1/6D)$, on the correlation function can be approximated by[8]

$$C(t) = C_0(t)\left[S^2 + (1 - S^2)\exp\left(-\frac{t}{\tau_e}\right)\right] \tag{29}$$

Fourier transformation of this correlation function provides the spectral density $J(\omega)$ function that determines the relaxation times. For anisotropic rotational diffusion and $\tau_e << (1/6D)$, J(ω) is expressed as:

$$J(\omega) = S^2 \sum\nolimits_{k=1,2,3} \frac{A_k\tau_k}{1 + (\omega\tau)^2} + \frac{(1 - S^2)\tau}{1 + (\omega\tau)^2} \tag{30}$$

With, $\tau = \tau_e/(6D\tau_e + 1)$.

Substituting equation S30 into S7 and using the following parameters, $k_B = 1.38065e^{-23}$ J/K, $\eta_0 = 8.9e^{-4}$ Pa.s (Viscosity of water at 298K), $T = 298$K, $L = 43.82$Å, $2R = 19.97$Å (obtained from pdbID 5uzd[9]), $S^2 = 0.8$ typical value for double stranded DNA[10], the ^{1}H $R_{1\rho}$ RD simulation using equation S17 revealed that the cross-relaxation effects are negligible for dipolar proton at distances ≥ 2.8Å. This is lower by 0.2Å compared to the 3Å distance observed from the simulations performed using isotropic spectral density function from equation S7. This put the 3Å distance in a conservative estimate thereby providing confidence in performing ^{1}H $R_{1\rho}$ RD experiments on nucleic acids provided it satisfies the conditions listed in the conclusion of the main text.

Supplementary Discussion

Effects of cross-relaxation on ^1H $R_{1\rho}$ RD

To analyse the effect of cross-relaxation on $R_{1\rho}$ RD profiles in the $R_{1\rho}$ experiment (Figure 2a), a model involving a two-state exchange between a GS and ES in the presence of neighbouring dipolar coupled proton ($^1H_{dip}$) was considered (Figure 2b). The $^1H_{dip}$ was positioned at distances of r_i and r_j relative to the GS and ES, respectively, which can be varied to consider multiple scenarios. Transverse (R_2) and longitudinal (R_1) auto-relaxation rates, together with chemical exchange, are described by the Bloch-McConnel (BM) equations (Supplementary equations 1–16)[1,2,11–13]. These can be expanded to include transverse (μ) and longitudinal (σ) cross-relaxation rates induced by $^1H_{dip}$ (Supplementary equation 17–18)[3]. The resulting BM matrix can be propagated over the pulse sequence "ramp ($\varphi = -Y$) – spin-lock strength (ω_{SL}) ($\varphi = X$) – ramp ($\varphi = Y$)" (Figure 2a), the main component of the $R_{1\rho}$ RD experiment[14,15]. The initial magnetization vector aligned along the z-axis was weighted using the populations of the GS (p_{GS}) and ES (p_{ES}) as specified during the simulation. For simplicity, the $^1H_{dip}$ magnetization was not weighted since it is assumed to be a static proton not undergoing any chemical exchange. Isotropic spectral density function, J(ω) (Supplementary equation 7), was calculated with a rotational correlation time (τ_c) of 5.1 ns based on the size of the A_2 DNA duplex[9].

$R_{1\rho}$ RD experiments can be performed by varying two parameters: (a) the spin-lock strength (ω_{SL}), which is positioned on-resonance at the observed chemical shift, and (b) the position of ω_{SL} known as the offset (Ω_{SL}) which is varied by approximately $\pm 4 \times \omega_{SL}$ relative to the observed chemical shift, to sample the resonance position of the ES[1]. The Z-magnetization (M_Z) under increasing duration of ω_{SL} (τ_{SL}) decays monoexponentially (Supplementary equation 4) with the rate constant $R_{1\rho}$. In the absence of $^1H_{dip}$ $R_{1\rho}$ is represented as (Supplementary equation 15)[2,13]. Accounting for the cross-relaxation Supplementary equation 15 can be extended to Supplementary equation 19. On-resonance, where $\theta = 90°$, the $R_{1\rho}$ rate approaches $R_2 + \mu$ in the high spin-lock limit while off-resonance, when $\Omega_{SL} >> \omega_{SL}$, it approaches $R_1 + \sigma$. Within this framework, the following scenarios were considered to discuss the effect of cross-relaxation on both on- and off-resonance experiments using common exchange parameters of k_{ex} = 2 kHz, p_{ES} = 0.5%, τ_c = 5.1 ns, $R_{1GS} = R_{1ES} = R_{1dip} = 2.5$ s^{-1}, $R_{2GS} = R_{2ES} = R_{2dip} = 22.5$ s^{-1}, $\Delta\omega_{ES}/2\pi = +600$ Hz and $\Delta\omega_{dip}/2\pi = -600$ Hz:

Scenario 1: Cross-relaxation only in the GS and not the ES

The distance r_i ($^1H_{dip}$ to GS proton) was varied from 2 Å to 4 Å, while cross-relaxation in the ES was neglected by setting r_j ($^1H_{dip}$ to ES proton) to a sufficiently large value (40 Å). This approach models a dynamic nucleic acid system, such as a base flipping out of the helical axis in the ES, which increases the distance between the proton of interest and $^1H_{dip}$ compared to the GS. Simulated on-resonance $R_{1\rho}$ (Figure 2c) reveals an increased μ contribution when r_i < 3 Å, corresponding to the ^{N}H1–^{N}H3 distance (2.3 Å) in GU/GT wobble base-pairs (Figure 1g). At distances r_i < 2.5 Å–typical of geminal protons such as H5', H5'' and H2', H2'' in the deoxyribose sugar and aromatic H6, H5 protons– $R_{1\rho}$ initially rises at ω_{SL} < 1 kHz and approaches the $R_2 + \mu$ in the high spin-lock limit (Supplementary Figure 1a). At $r_i \geq 3$ Å, which represents inter-imino proton distances in canonical B-form DNA and A-form RNA, the μ contribution remains minimal, contributing ≤ 5% to R_2 (Figure 2c).

The simulated off-resonance profile (Figure 2d) demonstrates that for $\Omega_{SL} >> \omega_{SL}$, $R_{1\rho}$ approaches $R_1 + \sigma$. At $r_i \leq 2.2$ Å, this sum becomes negative due to the dominant contribution of σ, leading to an exponential increase in Z-magnetization (Supplementary Figure 1b). This magnetization buildup complicates the estimation of $R_2 + R_{ex}$ and necessitates an approximate analytical solution beyond the scope of this study. For r_i between 2.2 and 2.5 Å and assuming $\Delta\omega_{dip} = -600$ Hz (1 ppm at a 600 MHz ^{1}H Larmour frequency used in this work), the $R_2 + R_{ex}$ profile exhibits a response from $^1H_{dip}$, characterized by a shift in local maximum of $R_2 + R_{ex}$ as ω_{SL} increases (Figure 2d, Supplementary Figure 1c). This behaviour, which distinguishes it from ES to GS exchange, diminishes at $r_i \geq 3$ Å. In contrast, the intra-base-pair protons, such as amino (-NH_2), aromatic (^{C}H2 and ^{C}H8), and imino (^{N}H3/H1) protons at distances $r_i \leq 3$Å (Figure 1d, 1e and 1g) do not exhibit this effect due to their large chemical shift differences of 2.1 to 2.7 kHz (−3.5 to −4.5 ppm), which lie beyond the conventionally probed Ω_{SL} range (±4x ω_{SL}). However, if Ω_{SL} is near $\Delta\omega_{dip}$, a response is observed (Supplementary Figure 1d).

Simulations varying k_{ex} and $\Delta\omega_{dip}$ (Supplementary Figures 1e – 1h) indicate minimal cross-relaxation effects at $r_i \geq 3$ Å and $|\Omega_{SL}| < |\Delta\omega_{dip}|$. Using the anisotropic definition of the spectral density function to account for the axial symmetry of the A_2 DNA (Supplementary equation 24 – 30), it was observed that the contribution from the cross relaxation is minimal at $r_i \geq 2.8$ Å (Figure S1i) which differs minimally from the simulations using isotropic spectral density function (equation S7). The impact of $\Delta\omega_{dip}$ on the ^{1}H $R_{1\rho}$ RD experiment was also evaluated. For $r_i = 2.5$ Å, an increase in on-resonance $R_{1\rho}$ rate is observed when $\Delta\omega_{dip}$ approaches the GS resonance (Supplementary Figures 2a – c). When $\Delta\omega_{dip}$ and $\Delta\omega_{ES}$ are in the same direction, accurate extraction of chemical exchange parameters becomes challenging due to difficulty in disentangling the contribution of exchange and cross-relaxation to $R_{1\rho}$ (Supplementary Figure 2b). For $r_i \geq 3.0$ Å, these effects are mitigated, enabling reliable extraction of exchange parameters.

Scenario 2 and 3: Cross-relaxation equal in both GS and ES or is larger in ES

In this scenario, chemical exchange involves an ES conformer susceptible to cross-relaxation, where the neighbouring $^1H_{dip}$ is positioned such that: (a) the $^1H_{dip}$ is equidistant to the proton of interest in both GS and ES (Figure 2e), or (b) the $^1H_{dip}$ is closer to the proton of interest in the ES compared to the GS (example: $r_j = r_i - 0.2$ Å) (Figure 2g). Simulations of on-resonance profiles reveal that the cross-relaxation contribution increases as the distance r_j decreases relative to r_i. The $R_{1\rho}$ rates approach $R_2 + \mu$ at $r_i = 3.3$ Å (within ± 5% error), compared to 3 Å in scenario 1 (Figure 2f, 2h). For $r_i < 2.4$ Å, $R_2 + \mu$ exceeds R_{ex}. This enhanced cross-relaxation also amplifies the exponential rise observed in the off-resonance profile under this scenario (Supplementary Figures 2d – g). Consistent with scenario 1, cross-relaxation effects are minimal at distances $r_i \geq 3$Å.

As a result, ^{1}H $R_{1\rho}$ RD provides a valuable tool for studying conformational exchange involving nucleobase protons, provided $\Delta\omega_{ES}$ lies within and $\Delta\omega_{dip}$ remains outside the probed Ω_{SL} range. Prior knowledge of neighbouring protons and chemical shifts can help in designing specifically labelled sample and estimate cross-relaxation effects.

Supplementary References

1. Rangadurai, A., Szymaski, E. S., Kimsey, I. J., Shi, H. & Al-Hashimi, H. M. Characterizing micro-to-millisecond chemical exchange in nucleic acids using off-resonance R1ρ relaxation dispersion. *Progress in Nuclear Magnetic Resonance Spectroscopy* **112–113**, 55–102 (2019).
2. Trott, O. & Palmer, A. G. R1ρ Relaxation outside of the Fast-Exchange Limit. *Journal of Magnetic Resonance* **154**, 157–160 (2002).
3. Allard, P., Helgstrand, M. & Härd, T. A Method for Simulation of NOESY, ROESY, and Off-Resonance ROESY Spectra. *Journal of Magnetic Resonance* **129**, 19–29 (1997).
4. Tjandra, N., Feller, S. E., Pastor, R. W. & Bax, A. Rotational diffusion anisotropy of human ubiquitin from 15N NMR relaxation. *J. Am. Chem. Soc.* **117**, 12562–12566 (1995).
5. Garcia De La Torre, J., Navarro, S. & Lopez Martinez, M. C. Hydrodynamic properties of a double-helical model for DNA. *Biophysical Journal* **66**, 1573–1579 (1994).
6. De La Torre, J. G. & Bloomfield, V. A. Hydrodynamic properties of complex, rigid, biological macromolecules: theory and applications. *Quart. Rev. Biophys.* **14**, 81–139 (1981).
7. Ortega, A. & García De La Torre, J. Hydrodynamic properties of rodlike and disklike particles in dilute solution. *The Journal of Chemical Physics* **119**, 9914–9919 (2003).
8. Lipari, G. & Szabo, A. Model-free approach to the interpretation of nuclear magnetic resonance relaxation in macromolecules. 1. Theory and range of validity. *J. Am. Chem. Soc.* **104**, 4546–4559 (1982).
9. Sathyamoorthy, B. *et al.* Insights into Watson–Crick/Hoogsteen breathing dynamics and damage repair from the solution structure and dynamic ensemble of DNA duplexes containing m1A. *Nucleic Acids Research* **45**, 5586–5601 (2017).
10. Furukawa, A., Walinda, E., Arita, K. & Sugase, K. Structural dynamics of double-stranded DNA with epigenome modification. *Nucleic Acids Res* **49**, 1152–1162 (2021).
11. Palmer, A. G. & Massi, F. Characterization of the Dynamics of Biomacromolecules Using Rotating-Frame Spin Relaxation NMR Spectroscopy. *Chem. Rev.* **106**, 1700–1719 (2006).
12. Massi, F., Johnson, E., Wang, C., Rance, M. & Palmer, A. G. NMR R1ρ Rotating-Frame Relaxation with Weak Radio Frequency Fields. *J. Am. Chem. Soc.* **126**, 2247–2256 (2004).
13. Miloushev, V. Z. & Palmer, A. G. R1ρ relaxation for two-site chemical exchange: General approximations and some exact solutions. *Journal of Magnetic Resonance* **177**, 221–227 (2005).
14. Steiner, E., Schlagnitweit, J., Lundstrçm, P. & Petzold, K. Capturing Excited States in the Fast-Intermediate Exchange Limit in Biological Systems Using 1H NMR Spectroscopy. *Angew. Chem.* 4 (2016).
15. Schlagnitweit, J., Steiner, E., Karlsson, H. & Petzold, K. Efficient Detection of Structure and Dynamics in Unlabeled RNAs: The SELOPE Approach. *Chemistry – A European Journal* **24**, 6067–6070 (2018).
16. Nikolova, E. N., Gottardo, F. L. & Al-Hashimi, H. M. Probing Transient Hoogsteen Hydrogen Bonds in Canonical Duplex DNA Using NMR Relaxation Dispersion and Single-Atom Substitution. *J. Am. Chem. Soc.* **134**, 3667–3670 (2012).
17. Zhou, H. *et al.* m1A and m1G disrupt A-RNA structure through the intrinsic instability of Hoogsteen base pairs. *Nat Struct Mol Biol* **23**, 803–810 (2016).
18. Schanda, P., Kupče, Ē. & Brutscher, B. SOFAST-HMQC Experiments for Recording Two-dimensional Deteronuclear Correlation Spectra of Proteins within a Few Seconds. *J Biomol NMR* **33**, 199–211 (2005).
19. Swails, J., Zhu, T., He, X. & Case, D. A. AFNMR: automated fragmentation quantum mechanical calculation of NMR chemical shifts for biomolecules. *J Biomol NMR* **63**, 125–139 (2015).
20. Nikolova, E. N. *et al.* Transient Hoogsteen base pairs in canonical duplex DNA. *Nature* **470**, 498–502 (2011).
21. Liu, B., Rangadurai, A., Shi, H. & Al-Hashimi, H. M. Rapid assessment of Watson–Crick to Hoogsteen exchange in unlabeled DNA duplexes using high-power SELOPE imino ^{1}H CEST. *Magnetic Resonance* **2**, 715–731 (2021).
22. Case, D. A. Using quantum chemistry to estimate chemical shifts in biomolecules. *Biophys Chem* **267**, 106476 (2020).
23. Zhu, T., Zhang, J. Z. H. & He, X. Automated Fragmentation QM/MM Calculation of Amide Proton Chemical Shifts in Proteins with Explicit Solvent Model. *J. Chem. Theory Comput.* **9**, 2104–2114 (2013).
24. Zhang, J. *et al.* Automated Fragmentation Quantum Mechanical Calculation of 15N and 13C Chemical Shifts in a Membrane Protein. *J. Chem. Theory Comput.* **19**, 7405–7422 (2023).
25. Palmer, A. G., Kroenke, C. D. & Patrick Loria, J. [10] - Nuclear Magnetic Resonance Methods for Quantifying Microsecond-to-Millisecond Motions in Biological Macromolecules. in *Methods in Enzymology* (eds James, T. L., Dötsch, V. & Schmitz, U.) vol. 339 204–238 (Academic Press, 2001).
26. Pérez de Alba Ortíz, A., Vreede, J. & Ensing, B. The Adaptive Path Collective Variable: A Versatile Biasing Approach to Compute the Average Transition Path and Free Energy of Molecular Transitions. in

Biomolecular Simulations: Methods and Protocols (eds Bonomi, M. & Camilloni, C.) 255–290 (Springer, New York, NY, 2019). doi:10.1007/978-1-4939-9608-7_11.

27. Ray, D. & Andricioaei, I. Free Energy Landscape and Conformational Kinetics of Hoogsteen Base Pairing in DNA vs. RNA. *Biophysical Journal* **119**, 1568–1579 (2020).
28. Chakraborty, D. & Wales, D. J. Energy Landscape and Pathways for Transitions between Watson–Crick and Hoogsteen Base Pairing in DNA. *J. Phys. Chem. Lett.* **9**, 229–241 (2018).
29. Vreede, J., Pérez de Alba Ortíz, A., Bolhuis, P. G. & Swenson, D. W. H. Atomistic insight into the kinetic pathways for Watson–Crick to Hoogsteen transitions in DNA. *Nucleic Acids Research* **47**, 11069–11076 (2019).
30. Geronimo, I. & De Vivo, M. Alchemical Free-Energy Calculations of Watson–Crick and Hoogsteen Base Pairing Interconversion in DNA. *J. Chem. Theory Comput.* **18**, 6966–6973 (2022).
31. Ortíz, A. P. de A., Vreede, J. & Ensing, B. Sequence dependence of transient Hoogsteen base pairing in DNA. *PLOS Computational Biology* **18**, e1010113 (2022).
