# Supplementary material for: 1H R1ρ relaxation identifies a hidden intermediate in DNA base-pairing
Source: Nat Commun. 2026 May 6;17:4114. doi: 10.1038/s41467-026-72559-6 (PMC13150003; doi:10.1038/s41467-026-72559-6)
Supplement: Supplementary file 2 — Description of Additional Supplementary Files [file 41467_2026_72559_MOESM2_ESM.pdf]

Description of Additional Supplementary Files

File Name: Supplementary Data 1
Description: Excel sheet consisting of ^{1}H R1ρ NMR fit parameters and statistical testing.
